# Desmoplakin and periplakin genetically and functionally contribute to eosinophilic esophagitis

**DOI:** 10.1038/s41467-021-26939-9

**Published:** 2021-11-23

**Authors:** Tetsuo Shoda, Kenneth M. Kaufman, Ting Wen, Julie M. Caldwell, Garrett A. Osswald, Pathre Purnima, Nives Zimmermann, Margaret H. Collins, Kira Rehn, Heather Foote, Michael D. Eby, Wenying Zhang, Netali Ben-Baruch Morgenstern, Adina Y. Ballaban, Jeff E. Habel, Leah C. Kottyan, J. Pablo Abonia, Vincent A. Mukkada, Philip E. Putnam, Lisa J. Martin, Marc E. Rothenberg

**Affiliations:** 1 Division of Allergy and Immunology, Cincinnati Children’s Hospital Medical Center, 3333 Burnet Ave, Cincinnati, OH 45229 USA; 2 Center for Autoimmune Genomics and Etiology, Cincinnati Children’s Hospital Medical Center, 3333 Burnet Ave, Cincinnati, OH 45229 USA; 3 Department of Pediatrics, University of Cincinnati College of Medicine, 3200 Burnet Avenue, Cincinnati, OH 45229 USA; 4 Department of Research, Cincinnati Veterans Affairs Medical Center, 3200 Vine St, Cincinnati, OH 45220 USA; 5 Division of Pathology, Cincinnati Children’s Hospital Medical Center, 3333 Burnet Ave, Cincinnati, OH 45229 USA; 6 Division of Human Genetics, Cincinnati Children’s Hospital Medical Center, 3333 Burnet Ave, Cincinnati, OH 45229 USA; 7 Division of Gastroenterology, Hepatology and Nutrition, Cincinnati Children’s Hospital Medical Center, 3333 Burnet Ave, Cincinnati, OH 45229 USA

**Keywords:** Medical genetics, Oesophagitis

## Abstract

Eosinophilic esophagitis (EoE) is a chronic allergic inflammatory disease with a complex underlying genetic etiology. Herein, we conduct whole-exome sequencing of a multigeneration EoE pedigree (discovery set) and 61 additional multiplex families with EoE (replication set). A series of rare, heterozygous, missense variants are identified in the genes encoding the desmosome-associated proteins DSP and PPL in 21% of the multiplex families. Esophageal biopsies from patients with these variants retain dilated intercellular spaces and decrease DSP and PPL expression even during disease remission. These variants affect barrier integrity, cell motility and RhoGTPase activity in esophageal epithelial cells and have increased susceptibility to calpain-14–mediated degradation. An acquired loss of esophageal DSP and PPL is present in non-familial EoE. Taken together, herein, we uncover a pathogenic role for desmosomal dysfunction in EoE, providing a deeper mechanistic understanding of tissue-specific allergic responses.

## Abstract (web summary)

Eosinophilic esophagitis (EoE) is a chronic allergic inflammatory disease with a complex underlying genetic etiology. Here, the authors identify a series of rare variants in DSP and PPL in multiplex families with EoE and uncover a pathogenic role for desmosomal dysfunction in EoE.

## Introduction

Eosinophilic esophagitis (EoE) is an emerging chronic allergic inflammatory esophageal disease clinically characterized by esophageal dysfunction; histologically by esophageal eosinophilia, epithelial hyperplasia, and dilated intercellular spaces (DIS) associated with impaired barrier function; and a high degree of heritability^1–3^. We have previously described high proband concordance in monozygotic twins (58%), substantiating a genetic etiology^4^. Most genetic studies have focused on analyzing common genetic variants by genome-wide association studies (GWAS), with evidence implicating the epithelial gene products calpain 14 (CAPN14) and thymic stromal lymphopoietin (TSLP), respectively^5–7^. Mechanistic studies have substantiated that CAPN14 contributes to impaired epithelial barrier function, mediated in part by lost expression of desmoglein 1 (DSG1)^8^, and that TSLP promotes adaptive type 2 T cell immunity associated with IL-5 and IL-13 overproduction^9^. Despite these advances, the causal gene variants and/or genomic networks for EoE pathogenesis remain unclear.

Common genetic variants in identified pathways only partially explain heritability of EoE. Odds ratios (OR) for previously associated single-nucleotide polymorphisms (SNPs) range from 0.76–2.57^1^, with the less frequent variants having larger effect sizes. These results suggest that rare variants are more likely to be disease-causing with moderate-to-large effect sizes. A challenge for GWAS, especially for rare conditions like EoE, is that rare variants are excluded due to power concerns^1^. In this study, we identify rare genetic variants conferring substantial risk for EoE by using a multiplex family-based study design and whole-exome sequencing (WES). We show the functionality of sequence variants by bioinformatics and experimental support at gene and variant levels following the guidelines of the National Human Genome Research Institute^10^.

## Results

### Candidate variants in EoE

**Fig. 1:**
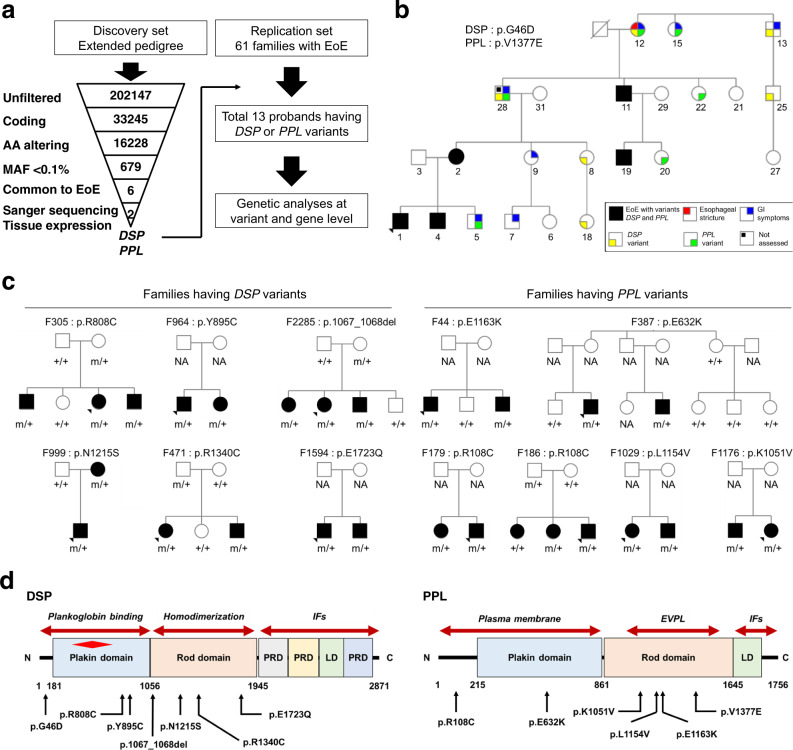
Identification of *DSP* and *PPL* variants by whole-exome sequencing. **a** Schema depicting the workflow for whole-exome sequencing (WES) filtering of rare variants in five patients with eosinophilic esophagitis (EoE) in the discovery set and confirmation in the replication set, as detailed in Methods. **b** Simplified pedigree of F430 (Details are in Supplementary Fig. 1). The arrowhead in the lower left corner indicates the proband and the slash indicates a deceased subject. “Not assessed” indicates the subject having GI symptoms but had never undergone an esophagogastroduodenoscopy. **c** Pedigrees of families with *DSP* or *PPL* variants in the replication set. Solid symbols indicate subjects with EoE, and open symbols indicate unaffected subjects. Arrowheads in the lower left corner indicate probands. For variant genotyping, “m” indicates the mutant *DSP* or *PPL* allele and “+” the reference allele. **d** Protein domain architectures and the location of amino acids predicted by mutations for *DSP* and *PPL*. The red diamond indicates a “hotspot” for mutations associated with cardiocutaneous disorders^30^. EoE eosinophilic esophagitis, *DSP* desmoplakin, *PPL* periplakin, AA amino acid, MAF minor allele frequency, GI gastrointestinal, NA not assessed, DSC1 desmocollin 1, JUP junction plakoglobin, PKP1 plakophilin 1, IFs intermediate filaments, EVPL envoplakin, PRD plakin repeat domain, LD linker domain.

The schematic workflow for the WES analysis is depicted in Fig. 1a. We initially conducted WES on the DNA from an extended pedigree (discovery set), which included five subjects who had histologically confirmed EoE and were across multiple generations (Fig. 1b and Supplementary Fig. 1). WES identified two coincident, heterozygous, missense variants in desmoplakin (*DSP*) and periplakin (*PPL*) [*DSP*: p.G46D (c.137 G → A), *PPL*: p.V1377E (c.4130 T → A)] (Fig. 1a–b) only in affected family members. Both *DSP* and *PPL* are members of the plakin protein family^11^ that localize to desmosomes and are highly expressed in the esophagus (Supplementary Table 1 and Supplementary Fig. 2). *DSP* and *PPL* are critical components of desmosome structures in epithelial cells, which are responsible for maintaining the structural integrity of cell contacts and cell migration^12,13^. Notably, *DSP* and *PPL* were expressed primarily in the squamous epithelium with strong relative expression in the esophageal mucosa (Supplementary Fig. 2). Subsequent Sanger sequencing of all 32 family members confirmed co-segregation of the two variants with each of the five affected members (Supplementary Fig. 1). Two additional members (#12 and #28) had both variants; #12 had a diagnosis of esophageal stricture, and #28 had reflux symptoms but had never undergone an esophagogastroduodenoscopy (EGD) (Fig. 1b). Potentially, both might have EoE. Additional family members had one variant, *DSP* or *PPL*. Co-segregation was found only in the variants in *DSP* and *PPL*, but not other candidate genes. The strong co-segregation of these variants supports a joint role of these variants in EoE etiology.

### Frequency of *DSP* and *PPL* mutations in EoE

We sequenced additional multiplex families (replication set), which included 61 index patients of European ancestry. In addition to the identified discovery variants (1 *DSP*, 1 *PPL*), we observed 6 *DSP* variants in six unique multiplex families (one variant per family) and 5 *PPL* variants in six other multiplex families (one variant shared by two families), which we confirmed by Sanger sequencing of all family members (genotype–phenotype concordance 78.3%, *P* = 0.005) (Fig. 1c). From the discovery and replication set, we identified a total of seven variants in *DSP* and six variants in *PPL* (Table 1, Fig. 1d, Supplementary Fig. 3–4), which were present in 13 of 62 families with EoE (21.0%). Only the discovery family had identified coincident *DSP* and *PPL* variants. These 13 variants of *DSP* and *PPL* are exceedingly rare based on the Exome Aggregation Consortium (ExAC)^14^, predicted to have a deleterious effect on protein function and evolutionarily conserved (Supplementary Tables 2–5). For *DSP* variants, the mutant amino acids are within the plakoglobin binding site, plakin domain and homodimerization site (Fig. 1d). For *PPL* variants, the mutant amino acids are within the plakin domain and the rod domain that forms complexes with envoplakin (Fig. 1d).

**Table 1:** Families with EoE and pathogenic variants in *DSP* and/or *PPL*.

Patient	Gender	Variant			Subject characteristics and medical history				
		Gene(s)	Protein	cDNA	Age at onset (years)	Atopic status	Peak Eos/hpf	Endoscopic phenotype	Symptoms
430^a^		*DSP*	p.G46D	c.G137A					
		*PPL*	p.V1377E	c.T4130A					
1	Male				8.1	BA, AR, AnFA	177	Inflammatory	Dysphagia, GERD, N/V, Pain
2	Female				45.0	BA	17	Normal-appearance	Dysphagia, GERD, N/V, Pain
4	Male				11.0	BA	55	Inflammatory	Dysphagia, GERD, Pain
11	Male				58.0	BA	73	Inflammatory	Dysphagia, GERD, N/V, Pain
19	Male				16.0	BA	24	Inflammatory	Dysphagia, GERD, N/V, Pain
305		*DSP*	p.R808C	c.C2422T					
1	Female				7.7	AR	145	Fibrostenotic	Dysphagia, GERD, N/V, Pain
2	Male				6.0	AR	154	Fibrostenotic	Dysphagia, GERD, N/V, Pain
6	Male				12.8	BA, AR	26	Fibrostenotic	Dysphagia, GERD, N/V, Pain
964		*DSP*	p.Y895C	c.A2684G					
1	Male				1.5	AR, AD, AnFA	65	Normal-appearance	Dysphagia, GERD, N/V, Pain
2	Female				4.1	AR, AD, AnFA	148	Inflammatory	Dysphagia, Pain
2285		*DSP*	p.1067_1068del	c.3201_3202del					
1	Female				5.5	BA, AR, AD	40	Inflammatory	Dysphagia, GERD, N/V, Pain
2	Female				4.0	BA, AR, AD	37	Inflammatory	Dysphagia, GERD, N/V, Pain
3	Male				2.5	BA, AR	41	Inflammatory	Dysphagia, GERD, N/V, Pain
999		*DSP*	p.N1215S	c.A3644G					
1	Male				2.6	AR, AD	100	Normal-appearance	Dysphagia, GERD, N/V, Pain
2	Female				33.2	AD	16	Inflammatory	Dysphagia, GERD, N/V, Pain
471		*DSP*	p.R1340C	c.C4018T					
1	Female				6.8	BA, AnFA	162	Fibrostenotic	Dysphagia, GERD, N/V, Pain
4	Male				8.6	BA, AR	134	Fibrostenotic	Dysphagia, GERD, N/V, Pain
1594		*DSP*	p.E1723Q	c.G5167C					
1	Male				15.5	BA, AR	60	Inflammatory	Dysphagia, GERD
2	Male				11.4	No	112	Fibrostenotic	Dysphagia, GERD, N/V, Pain
179		*PPL*	p.R108C	c.C322T					
1	Female				4.2	AR	147	Inflammatory	GERD, N/V, Pain
2	Male				3.1	AD	120	Inflammatory	GERD, N/V, Pain
186		*PPL*	p.R108C	c.C322T					
1	Male				1.0	No	155	Inflammatory	GERD, N/V, Pain
4	Female				12.0	BA, AR	332	Inflammatory	Dysphagia, GERD
387		*PPL*	p.E632K	c.G1894A					
1	Male				3.8	BA, AD, AnFA	33	Inflammatory	Dysphagia, GERD, N/V, Pain
2	Male				14.3	AR	50	Inflammatory	Dysphagia, GERD, N/V
1176		*PPL*	p.K1051V	c.3151_3152delinsGT					
1	Male				5.3	BA, AD	52	Inflammatory	Dysphagia, Pain
2	Female				3.5	BA, AD, AnFA	55	Fibrostenotic	Dysphagia, GERD, N/V, Pain
1029		*PPL*	p.L1154V	c.C3460G					
1	Female				8.1	BA, AR, AnFA	243	Inflammatory	Dysphagia, N/V, Pain
2	Male				8.6	BA, AR	137	Fibrostenotic	N/V, Pain
44		*PPL*	p.E1163K	c.G3487A					
1	Male				13.0	BA, AR, AnFA	54	Normal-appearance	Pain
4	Male				11.9	BA, AR, AnFA	95	Inflammatory	Pain

^a^ Discovery set *DSP* desmoplakin, *PPL* periplakin, BA bronchial asthma, AR allergic rhinitis, AnFA anaphylaxis by food, GERD gastroesophageal reflux, N/V nausea and/or vomiting, eos/hpf eosinophils per high-power microscopic field.

To evaluate whether these rare variants in *DSP* and *PPL* were enriched in EoE compared to controls, we examined rare genetic burden tests by case-control association studies with the use of ExAC. At the variant level, three of the 13 variants were not in the ExAC database, and eight reached statistical significance (Table S6). At the gene level, the frequency of *DSP* or *PPL* variants in the index patients from the 62 EoE multiplex families were higher than that of a control cohort of European ancestry from the ExAC database (*P* = 0.0021, Fisher’s exact test) (Table 2). This result was replicated by an independent control cohort from the UK Biobank^15^ (*P* = 0.023, Fisher’s exact test) (Table 2). Although we found enrichment of rare variants in these genes, we did not see the joint segregation of those two variants in the replication set. Given the fact that *DSP* and *PPL* variants were seen in the control cohorts, we speculate that isolated *DSP* and *PPL* variants are not sufficient for the disease but cooperate with secondary hits in the desmosomal genes. In the case of the discovery family, the second hit is the co-occurrence of damaging variants in both *DSP* and *PPL*, whereas, in the replication families, the co-occurrence of the *DSP* or *PPL* variants with additional rare variants in other genes in the desmosome were enriched (*P* = 0.0052, Fisher’s exact test) (Table 2 and S7), providing evidence that rare genetic variants in the desmosomal genes associate with EoE.

**Table 2:** *DSP* or *PPL* rare variant burden analysis in eosinophilic esophagitis (EoE).

Exome Aggregation Consortium (ExAC)^a^					
	Multiplex EoE cases (*n* = 62)		Controls (*n* = 33,370)		*P*-value (Fisher’s exact test)
	Minor allele	Major allele	Minor allele	Major allele	
*DSP* or *PPL*	14	234	3079	13,0401	0.0021

UK Biobank^b^					
	Multiplex EoE cases (*n* = 62)		Controls (*n* = 4,822)		
	Subjects with variants	Subjects without variants	Subjects with variants	Subjects without variants	*P*-value (Fisher’s exact test)
*DSP* or *PPL*	13	49	532	4,290	0.023
*DSP* or *PPL* with other rare variants in desmosome (GO:0030057)	8	54	206	4,616	0.0052

*EoE* eosinophilic esophagitis, *ExAC* Exome Aggregation Consortium, *UK* United Kingdom, *GO* gene ontology. Two-tailed *P*-values were determined by the Fisher’s exact test.

^a^Individuals of European ancestry from ExAC Database (https://gnomad.broadinstitute.org/). Observed allele counts were based on two alleles of each gene.

^b^Non-EoE individuals of European ancestry from the UK Biobank (https://www.ukbiobank.ac.uk/).

### Esophageal *DSP* and *PPL* expression and associated features in EoE

**Fig. 2:**
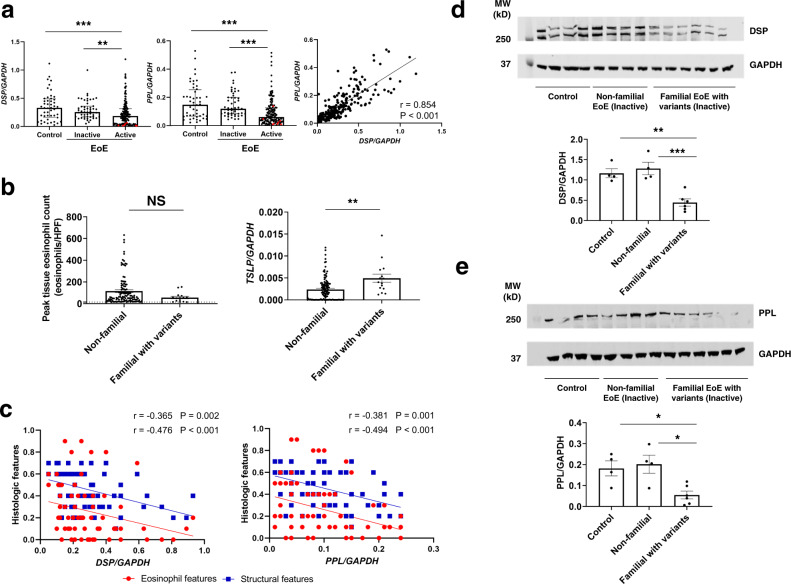
Expression of *DSP* and *PPL* in patients with EoE. **a** *DSP* (left) and *PPL* (middle) mRNA expression in esophageal biopsies from controls and patients with non-familial EoE (inactive and active). Each point represents an individual subject [(Control, *n* = 48; Inactive EoE, *n* = 51; Active EoE, *n* = 147); red data points represent patients with *DSP* or *PPL* variants (not included in the statistics)]. Statistics: *DSP* (Control vs. Inactive EoE, *P* > 0.9999; Control vs. Active EoE, *P* = 0.0007; Inactive EoE vs. Active EoE, *P* = 0.009); *PPL* (Control vs. Inactive EoE, *P* > 0.9999; Control vs. Active EoE, *P* < 0.0001; Inactive EoE vs. Active EoE, *P* < 0.0001). Correlation plot of *DSP* and *PPL* mRNA expression is also shown (right) (*n* = 246). Statistics: *P* < 0.0001. **b** Peak esophageal eosinophil counts (left) and *TSLP* mRNA expression (right) are plotted by groups for non-familial and familial EoE with *DSP* or *PPL* variants (non-familial EoE, *n* = 115; familial EoE with variants, *n* = 15). All samples were from the biopsies during the active disease state. The dashed line indicates the diagnostic threshold of EoE (15 eosinophil/hpf). Statistics: peak esophageal eosinophil count, *P* = 0.0927; *TSLP*, *P* = 0.0010. **c** Correlation plots of gene expressions (left: *DSP*, right: *PPL*) and histologic scores (red: eosinophil features, blue: structural features) (*n* = 68). Statistics: left (*DSP* with eosinophil features, *P* = 0.0022; *DSP* with structural features, *P* < 0.0001), right (*PPL* with eosinophil features, *P* = 0.0014; *PPL* with structural features, *P* < 0.0001). **d** and **e** Representative western blot analysis of DSP (**d**) and PPL (**e**) among control individuals (*n* = 4), patients with non-familial EoE (*n* = 4), and patients with familial EoE with *DSP* or *PPL* variants (*n* = 6). GAPDH serves as a loading control. For patients with EoE, all samples were from the biopsies during the inactive disease state. Statistics: **d** (control vs. non-familial EoE, *P* = 0.7833; control vs. familial EoE, *P* = 0.0022; non-familial EoE vs. familial EoE, *P* = 0.0007), **e** (control vs. non-familial EoE, *P* = 0.9061; control vs. familial EoE, *P* = 0.0317; non-familial EoE vs. familial EoE, *P* = 0.0142). For panels **a** (left and middle), **b, d** and **e**, data are presented as mean ± SEM. For panels **a**–**e**, n is the number of biologically independent subjects. For panels **a**–**e**, two-tailed *P*-values were determined by the following tests: **a** (left and middle), Kruskal–Wallis test followed by a Dunn multiple-comparison test; **b**, the unpaired *t*-test; **a** (right) and **c**, Spearman’s rank correlation coefficient (multiple comparisons were not applied); and **d**, **e**, one-way ANOVA test followed by a Tukey’s multiple comparisons test. *P < 0.05, **P < 0.01 and ***P < 0.001. EoE eosinophilic esophagitis, *DSP* desmoplakin, *PPL* periplakin, GAPDH Glyceraldehyde 3-phosphate dehydrogenase, hpf high-power microscopic field, IQR interquartile range, DAPI 4',6-diamidino-2-phenylindole, MW molecular weight, NS not significant, SEM standard error of the mean.

To obtain further in vivo evidence of the relevance of EoE-associated mutations to disease manifestations, esophageal biopsy specimens obtained from familial EoE (62 multiplex families), non-familial EoE (511 non-multiplex families), and controls (93 individuals) (Table S8) were analyzed using approaches including quantifying peak esophageal eosinophil counts, EoE histology scoring system (HSS), EoE diagnostic panel (EDP), single-cell RNA sequencing and immunofluorescence staining^16–18^. Esophageal *DSP* and *PPL* expression were markedly lower in patients with active EoE than inactive EoE and controls (Fig. 2a), as was the expression of genes encoding other desmosomal proteins, such as *DSG1* (Supplementary Fig. 5a). Among patients with active EoE having similar esophageal eosinophil levels, familial EoE with an identified *DSP* or *PPL* variant had significantly lower *DSP* or *PPL* expression, respectively, than did non-familial EoE (Supplementary Fig. 5b). *DSP* and *PPL* expression correlated with each other (*r* = 0.854, *P* < 0.001) (Fig. 2a). Significant correlations were noted between each gene (*DSP* or *PPL*) and several genes involved in EoE, especially downregulated genes (Supplementary Fig. 5c). *DSP* expression is inversely correlated with *TSLP* expression, which is notable because reduced *DSP* mediates impaired barrier function, which is an upstream event in the induction of *TSLP*^19^. *TSLP* is overexpressed in EoE and genetically and functionally linked with EoE^5,9^. In this regard, it is also notable that patients with familial EoE harboring *DSP* or *PPL* variants expressed higher *TSLP* mRNA levels than did patients with non-familial EoE (2.1 fold, *P* = 0.001) (Fig. 2b); the difference in *TSLP* expression was independent of peak eosinophil levels in the esophagus. These data support a possible relationship between desmosome variants and the type 2 skewing and allergic responses observed in EoE.

With regard to EoE histologic features, *DSP* and *PPL* expression inversely correlated with structural features and eosinophilic features (Fig. 2c, Supplementary Fig. 6a for non-familial EoE). Of note, non-familial EoE showed histologic changes that varied with the severity of disease activity, whereas familial EoE demonstrated retained structural features, mainly driven by DIS, regardless of disease activity (Table S9). This differential histology between non-familial and familial EoE was independent of esophageal eosinophil levels—peak esophageal eosinophil counts were not significantly different between non-familial and familial inactive EoE nor between non-familial and familial active EoE (Supplementary Fig. 6b–c). These findings suggest that decreased *DSP* and/or *PPL* expression, via the presence of *DSP* and/or *PPL* variants in familial EoE, impairs the epithelial barrier and causes fixed histologic features independent of esophageal eosinophilia. Immunofluorescence staining revealed that *DSP* is highly expressed in the basal and differentiating epithelial cells, whereas *PPL* is highly expressed in the suprabasal and differentiated epithelial cells (Supplementary Fig. 6d–g). Notably, DSP and PPL protein expression were reduced in esophageal biopsies obtained from patients with *DSP* and/or *PPL* variants, respectively, regardless of disease activity (Fig. 2d, e and Supplementary Fig. 6d–g). Single-cell RNA sequencing analysis of human esophageal biopsies also revealed co-expression of *DSP* and *PPL* at the single epithelial cell level (Supplementary Fig. 7) in some of the epithelial cells.

### *DSP* and *PPL* deficiency in esophageal epithelial cells

To uncover the role of *DSP* and *PPL* in esophageal epithelial homeostasis, we generated *DSP*- and *PPL*-deficient human esophageal cell lines (EPC2 cells) via clustered regularly interspaced short palindromic repeats (CRISPR)/Cas9-mediated genome editing. We examined the effect of *DSP* and *PPL* deletion on the barrier integrity following air–liquid interface (ALI) culture (Supplementary Fig. 8a). *DSP* and *PPL* knockout (KO) cells demonstrated acantholysis and significantly decreased barrier function, the latter determined by reduced transepithelial electrical resistance (TEER) and increased permeability to fluorescein isothiocyanate (FITC)-dextran compared to control cells (Supplementary Fig. 8b–d). Moreover, *DSP* KO cells and *PPL* KO cells showed increased migratory ability following a physical cellular injury (Supplementary Fig. 8e). Taken together, *DSP* or *PPL* loss in esophageal epithelial cells modifies cell motility and barrier integrity.

### Consequences of EoE-associated *DSP* and *PPL* variants

**Fig. 3:**
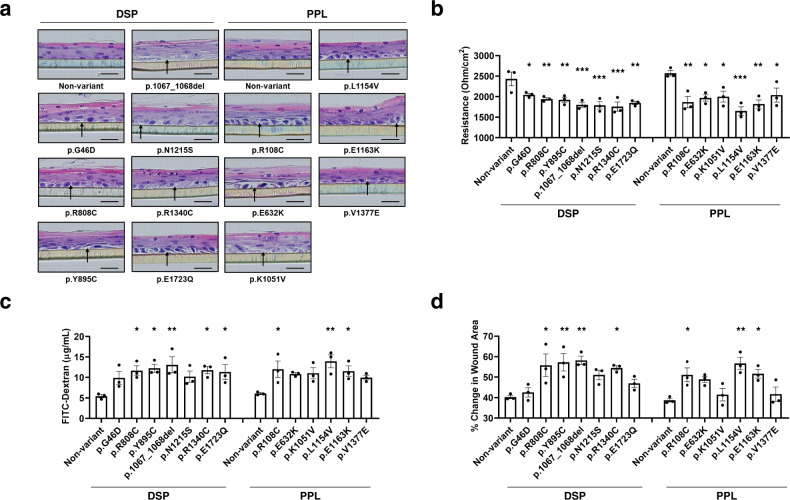
Effects of EoE-associated *DSP* and *PPL* variants. **a** Representative hematoxylin and eosin (H&E)–stained sections of EPC2 cells stably transduced with constructs encoding non-variant and mutated *DSP* or *PPL* after air–liquid interface (ALI) differentiation (day 14). Arrows point to the non-cellular areas that were formed. Scale bar: 50 μM. Data are representative of three experiments performed in duplicate. **b** The transepithelial electrical resistance (TEER) and **c** FITC-dextran flux measurements are shown for EPC2 cells grown at the ALI. **d** Wound healing assays performed in EPC2 cells transduced with constructs encoding non-variant and mutated *DSP* or *PPL*. Quantification of the wound closure after 8 h was shown. For panels **b**–**d** data are representative of three experiments performed in duplicate and are presented as mean ± SEM. Statistics (versus non-variants): **b** (p.G46D, *P* = 0.0324; p.R808C, *P* = 0.0059; p.Y895C, *P* = 0.0042; p.1067_1068del, *P* = 0.0006; p.N1215S, *P* = 0.0005; p.R1340C, *P* = 0.0003; p.E1723Q, *P* = 0.0012; p.R108C, *P* = 0.0041; p.E632K, *P* = 0.0131; p.K1051V, *P* = 0.0189; p.L1154V, *P* = 0.0004; p.E1163K, *P* = 0.0024; p.V1377E, *P* = 0.0291), **c** (p.G46D, *P* = 0.1542; p.R808C, *P* = 0.0274; p.Y895C, *P* = 0.0151; p.1067_1068del, *P* = 0.0062; p.N1215S, *P* = 0.1189; p.R1340C, *P* = 0.0244; p.E1723Q, *P* = 0.0385; p.R108C, *P* = 0.0187; p.E632K, *P* = 0.066; p.K1051V, *P* = 0.0536; p.L1154V, *P* = 0.0022; p.E1163K, *P* = 0.0304; p.V1377E, *P* = 0.1601) and **d** (p.G46D, *P* = 0.9917; p.R808C, *P* = 0.0103; p.Y895C, *P* = 0.005; p.1067_1068del, *P* = 0.0031; p.N1215S, *P* = 0.0944; p.R1340C, *P* = 0.0187; p.E1723Q, *P* = 0.4656; p.R108C, *P* = 0.0249; p.E632K, *P* = 0.0748; p.K1051V, *P* = 0.9471; p.L1154V, *P* = 0.0016; p.E1163K, *P* = 0.0194; p.V1377E, *P* = 0.9261). For panels **b**–**d**, two-tailed *P*-values were determined by the one-way ANOVA test followed by a Dunnett’s multiple-comparison test. *P < 0.05, **P < 0.01, and ***P < 0.001. EoE eosinophilic esophagitis, *DSP* desmoplakin, *PPL* periplakin, FITC fluorescein isothiocyanate, SEM standard error of the mean.

To further investigate the consequences of the EoE-associated missense variants in *DSP* and *PPL*, we transduced EPC2 cells with constructs encoding non-variant and mutated variant *DSP* and *PPL*. Immunoblotting of the cell extracts showed that expression of all mutant *DSP* and *PPL* constructs were equivalent to that of the wild-type construct (Supplementary Fig. 9). We examined the effect of *DSP* and *PPL* variants on the barrier integrity following epithelial culture at the ALI. Impaired epithelial barrier integrity and reduced barrier function were observed in cells transduced with the mutated variants compared with the wild-type *DSP* and *PPL* (Fig. 3a–c).

**Fig. 4:**
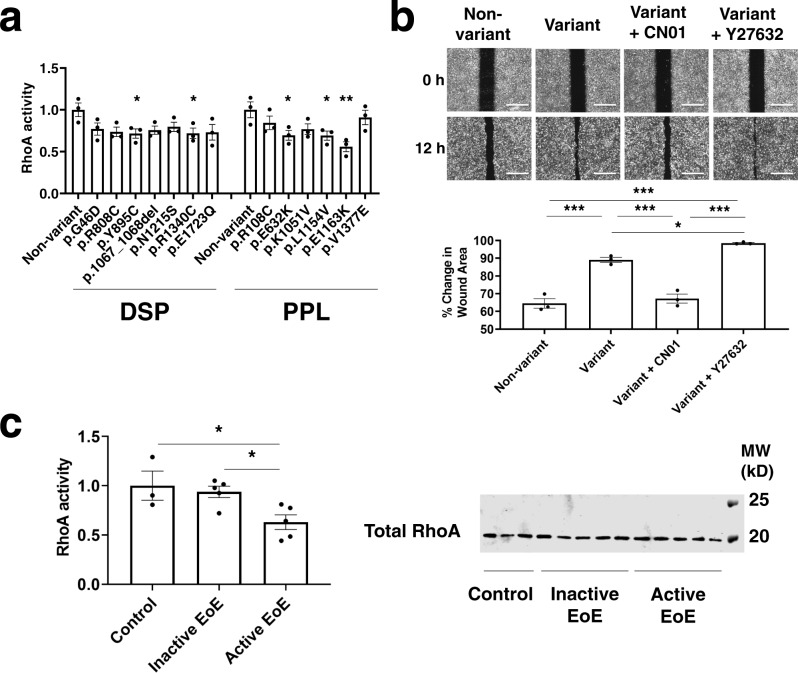
Effect of *DSP* and *PPL* variants on RhoA activity. **a** Active RhoA assays performed in EPC2 cells transduced with constructs encoding non-variant and mutated DSP or PPL. Statistics (versus non-variants): p.G46D, *P* = 0.132; p.R808C, *P* = 0.0691; p.Y895C, *P* = 0.0453; p.1067_1068del, *P* = 0.1013; p.N1215S, *P* = 0.2131; p.R1340C, *P* = 0.048; p.E1723Q, *P* = 0.0619; p.R108C, *P* = 0.4849; p.E632K, *P* = 0.0443; p.K1051V, *P* = 0.1589; p.L1154V, *P* = 0.0419; p.E1163K, *P* = 0.0035; p.V1377E, *P* = 0.8738. **b** Wound healing assays performed in EPC2 cells transduced with constructs encoding non-variant and mutated *DSP* (p.Y895C) treated with the Rho activator CN01 or the Rho kinase inhibitor Y27632. Quantification of the wound closure after 12 h was shown. Scale bar: 500 μM. Statistics: Non-variant vs. Variant, *P* = 0.0001; Non-variant vs. Variant + CN01, *P* = 0.7658; Non-variant vs. Variant + Y27632, *P* < 0.0001; Variant vs. Variant + CN01, *P* = 0.0002; Variant vs. Variant + Y27632, *P* = 0.0394; Variant + CN01 vs. Variant + Y27632, *P* < 0.0001. **c** Enzyme-linked immunosorbent assay of the level of active RhoA (RhoA-GTP) in protein lysates of biopsies from control individuals, non-familial patients with inactive EoE and non-familial patients with active EoE (Control, *n* = 3; Inactive EoE; *n* = 3; Active EoE, *n* = 5). Western blot analysis shows the expression of total RhoA in protein lysates of biopsies from each subject. Statistics: control vs. inactive EoE, *P* > 0.8803; control vs. active EoE, *P* = 0.0398; inactive EoE vs. active EoE, *P* = 0.0479. For panels **a**, **b**, data are representative of three experiments performed in duplicate and are presented as mean ± SEM. For panel **c**, data are presented as mean ± SEM, with markers representing biologically independent subjects. For panels **a**–**c**, two-tailed *P*-values were determined by the following tests: the one-way ANOVA test followed by a Dunnett’s multiple-comparison test (**a**) or Tukey’s multiple comparisons test (**b**, **c**). *P < 0.05, **P < 0.01, and ***P < 0.001. *DSP* desmoplakin, *PPL* periplakin, MW molecular weight, SEM standard error of the mean.

We assessed epithelial cell reconstitution using a wound healing assay and found that cells transduced with the mutated variants closed the wound faster than did control cells transduced with wild-type *DSP* and *PPL* (Fig. 3d and Supplementary Fig. 10). Notably, similar to mutated variants in this study, partial gene silencing and some of the known *DSP* mutations [p.R451G from arrhythmogenic cardiomyopathy and p.H586P from severe dermatitis, multiple allergies, and metabolic wasting (SAM) syndrome]^20,21^ also showed increased cell motility (Supplementary Fig. 10b and d). In non-esophageal keratinocytes (HaCaT cells), there were similar mutational effects, although variability was observed (Supplementary Fig. 10d, e). Regarding a potential mechanism, the observed effects on cell motility in the wound healing assay were suggestive of aberrant RhoGTPase signaling^22^, especially because it was reported that DSP knockdown cells have lower RhoA activity^23^. To investigate whether desmosomal variants affect RhoGTPase signaling, we measured levels of GTP-bound RhoA (active RhoA). Of note, RhoA activity was reduced in most of the cells transfected with variant *DSP* or *PPL* compared to cells transfected with wild-type versions of these genes (Fig. 4a). Additionally, restoration of Rho activity (using the Rho activator calpeptin) rescued the enhanced migration. Furthermore, blocking Rho signaling (via the Rho kinase inhibitor Y27632) further increased migration (Fig. 4b). Taken together, the RhoA pathway is likely associated with the *DSP* and *PPL* mutational effects. Indeed, we also demonstrated decreased active RhoA in biopsies from active EoE compared to that of inactive EoE and normal controls (Fig. 4c). Taken together, these data suggest that loss of function in epithelial homeostasis (by functional haploinsufficiency or dominant-negative effects of overexpressed mutant protein) is likely a relevant pathogenic mechanism.

### Calpain 14–mediated desmosomal protein degradation

**Fig. 5:**
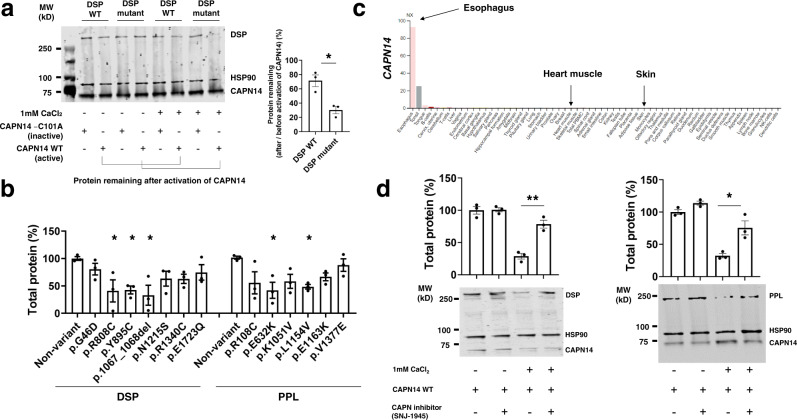
Calpain 14–mediated desmosomal protein degradation. **a** Immunoblots of lysates from *DSP* (non-variant or mutant p.Y895C)–transfected HEK293T cells with CAPN14 or enzymatically inactive CAPN14-C101A co-transfection for changes in *DSP* levels following the addition of exogenous Ca^2+^ (left). Protein remaining after activation of CAPN14 was determined by the difference in band intensity between after and before activation [(after x 100)/before] (right). Statistics: *DSP* WT vs. *DSP* mutant, *P* = 0.0131. **b** Effect of *DSP* and *PPL* variants on protein degradation on activation of co-transfected CAPN14. Total protein remaining was determined for each mutation. Total protein (%) = (each protein remaining x 100)/average of non-variant cells. Statistics (versus non-variants): p.G46D, *P* = 0.8212; p.R808C, *P* = 0.0296; p.Y895C, *P* = 0.0352; p.1067_1068del, *P* = 0.0125; p.N1215S, *P* = 0.2675; p.R1340C, *P* = 0.2581; p.E1723Q, *P* = 0.6186; p.R108C, *P* = 0.0712; p.E632K, *P* = 0.0156; p.K1051V, *P* = 0.0946; p.L1154V, *P* = 0.0323; p.E1163K, *P* = 0.2167; p.V1377E, *P* = 0.9157. **c** Gene expression of *CAPN14* in human normal tissues from the Human Protein Atlas (https://www.proteinatlas.org/). **d** Calpain inhibition results in rescue of *DSP* and *PPL* levels by CAPN14-mediated degradation. Total protein (%) = (each protein remaining x 100)/average of cells without exogenous Ca^2+^ and SNJ-1945. Statistics: *DSP* (without SNJ-1945 vs. with SNJ-1945, *P* = 0.0028); *PPL* (without SNJ-1945 vs. with SNJ-1945, *P* = 0.0188). For panels **a**, **b**, and **d**, data are representative of three experiments performed in duplicate and are presented as mean ± SEM, and two-tailed *P*-values were determined by the unpaired *t*-test (**a** and **d**) or one-way ANOVA test followed by a Dunnett’s multiple-comparison test (**b**). *P < 0.05 and **P < 0.01. CAPN14 calpain-14, *DSP* desmoplakin, PPL periplakin, SEM standard error of the mean, WT wild-type.

The identified *DSP* and *PPL* variants associated with EoE are distinct from the pathogenic variants associated with other phenotypes such as arrhythmias^24^. We aimed to understand the reason for the non-redundant phenotypes, i.e., esophageal but not other tissue involvement, in the EoE multiplex families having *DSP* and *PPL* variants. Of note, although we primarily observed missense mutations associated with EoE, more severe truncating mutations of *DSP* and *PPL* are associated with skin and heart diseases^25–27^. Thus, we hypothesized that the identified genetic variants might interact with tissue-specific pathways through a post-translational mechanism. We have previously identified CAPN14 as an esophageal-specific functional protease that induces esophageal epithelial barrier impairment and loss of DSG1 expression, likely by a degradative mechanism^6,8^. In this context, we hypothesized that cells carrying the *DSP* or *PPL* genetic variants might be more susceptible to CAPN14 proteolysis than cells carrying wild-type DSP or PPL. In order to test this hypothesis, we co-transfected expression vectors encoding CAPN14 with *DSP* (wild-type and mutant) or with *PPL* (wild-type and mutant). Cellular lysates were subsequently incubated with calcium to activate endogenous calpain; both *DSP* and PPL were degraded by co-transfection with CAPN14, but the degradation was less with the enzymatically inactive CAPN14-C101A, supporting that CAPN14 proteolysis contributes to this degradation (Fig. 5a). Of note, *DSP* and *PPL* mutants showed increased degradation compared to wild type (Fig. 5b). Considering the preferential expression of *CAPN14* in esophageal mucosa (Fig. 5c) and its strong association with EoE^28^, these results provide a potential mechanistic framework to understand the functional significance of the *DSP* and *PPL* mutants, as well as a potential mechanism that may link the observed esophagus-specific phenotype (compared to skin or heart disease phenotypes). Notably, *CAPN14* mRNA expression in esophageal biopsies showed a trend of being lower in patients with familial EoE than non-familial EoE (*P* = 0.23) (Supplementary Fig. 11a). However, there was no difference in the allele frequency of a *CAPN14*-tagging SNP between families with or without *DSP* and/or *PPL* rare variants (Supplementary Fig. 11b). Finally, we hypothesized that inhibiting calpain activity would ameliorate the increased CAPN14-mediated degradation. We focused our attention on a pharmacologic drug with broad calpain inhibitory activity, using SNJ-1945, an agent designed to have high water solubility and bioavailability^29^. Indeed, SNJ-1945 rescued cleavage by CAPN14 at least in part (Fig. 5d). Taken together, these findings provide a deeper molecular understanding of EoE pathogenesis and substantiate the potential therapeutic value of calpain inhibitors in modifying the identified genetic pathway.

## Discussion

Our study advances the field by identifying a pathogenic role for desmosomal dysfunction in EoE and the likely intersection of this dysfunction with calpain-14 and RhoGTPase–mediated pathways. More specifically, we identified *DSP* and *PPL* variants as likely being functionally involved in disease pathogenesis, as substantiated by multiple lines of evidence. First, *DSP* and *PPL* variants, encoding desmosomal proteins, strongly segregated in a multigenerational EoE pedigree (discovery) and were enriched in 21% of EoE multiplex families. Second, a series of functional analyses using an organotypic-like ALI culture system demonstrated that modulating wild-type *DSP* and *PPL* expression in vitro was functionally sufficient to induce changes in epithelial integrity (e.g., acantholysis) and barrier impairment, processes that are dysregulated in EoE^30,31^. Third, we determined that the identified *DSP* and *PPL* variants encode for proteins that have enhanced susceptibility to degradation by calpain-14, an esophageal specific protease that is involved in disease pathogenesis (based on GWAS, epigenetic and functional studies)^6,8,32^, thereby providing a pathogenic mechanism for the previously unexplained tissue-specific nature of this condition. Fourth, expression of the identified *DSP* and *PPL* variants modified epithelial motility in a wound model and mechanistically affected RhoGTPase signaling. Fifth, we observed that *DSP* and *PPL* loss occurs in non-familial EoE, substantiating that the pathway identified initially by rare familial EoE cases is broadly applicable to familial and non-familial EoE. We present evidence that non-familial EoE likely has an acquired dysregulation (differential *DSP* and *PPL* expression on the basis of disease activity), whereas the defect in multiplex families appears to be fixed, as demonstrated by the retained DIS and decreased *DSP* and *PPL* expression even during disease remission. Sixth, we have found that familial EoE harboring *DSP* or *PPL* variants express higher *TSLP* mRNA levels than does non-familial EoE, providing a mechanistic link between desmosome variants and the observed type 2 immune skewing and allergic responses in EoE. This is particularly interesting as common genetic variants in *TSLP* confer susceptibility to EoE^5,9^.

Our results mechanistically explain the previous findings of abnormal desmosome structure and barrier protein expression and impaired barrier function in biopsy specimens of patients with EoE^16,33,34^. Previous observations demonstrated that loss of desmosomal proteins results in a spectrum of epithelial integrity defects depending on the severity of disruption^12^. This is most evident in humans, as a large number of mutations covering most desmosomal genes have been described^35,36^. For instance, homozygous mutations in the epithelial adhesion molecule DSG1 cause SAM syndrome and are associated with EoE^36,37^. Multiple studies have emphasized that the inherited and acquired defects of plakins (i.e., DSP or PPL) in humans and animal models potentially lead to dramatic manifestations in the epithelium^13,35,38^, which is consistent with our mechanistic in vitro findings and clinical findings of EoE. Thus, the decreased DSP and PPL expression identified herein may exert a deleterious effect on epithelial homeostasis, which underlies the disease phenotype seen in EoE.

Besides the impact of activated eosinophils as a functional endpoint, a growing body of evidence supports a role for altered barrier function in EoE. The histopathologic hallmark of EoE is the presence of eosinophils in a hyperplastic esophageal epithelium. Dysregulation of intercellular junctions suggests a mechanism whereby inflamed and hyperproliferative esophageal epithelial tissue can be penetrated by allergens, thereby amplifying allergic inflammation, although whether these alterations in the epithelia represent a primary causative feature or a reactive secondary event following eosinophilic inflammation is uncertain due to disease heterogeneity in EoE. The genetic evidence presented herein supports a primary role for desmosomal dysfunction in EoE, at least in the identified familial cases.

It is notable that individuals with familial EoE with *DSP*/*PPL* variants had low peak eosinophil counts even during disease remission, which is similar to individuals with non-familial EoE. However, inactive EoE was defined by the eosinophil counts (<15 eosinophils/HPF), which may be insufficient to fully characterize the disease state or differentiate subtle mechanisms or outcomes of familial EoE during disease remission. For instance, although anti-inflammatory therapy (e.g., steroids) and elimination of causal food allergens can decrease eosinophil counts, subjects with *DSP*/*PPL* variants may maintain a basal level of “leakiness”. Thus, more intense therapy may be required in patients harboring the identified genetic variants. Notably, there were stronger relationships between *DSP* and *PPL* expression and structural features than eosinophil features in non-familial EoE. In familial EoE, we demonstrated a specific disruption of structural features in the esophageal epithelium of patients with *DSP* and *PPL* variants regardless of disease activity status, suggesting that barrier dysfunction occurred as a primary fixed defect. As we did not have detailed clinical data, it is unclear whether *DSP* and *PPL* variants may contribute to disease severity and/or treatment responses; a further characterization of clinical features will be a valuable future endeavor.

A challenge in the field has been explaining how a generalized impaired barrier function can result in the tissue-specific nature of EoE. Though *DSP* and *PPL* are highly expressed in the esophagus, they are also abundant in some tissues that experience mechanical stress, such as the skin and myocardium. Previous reports have shown that pathogenic mutations are associated with clinical phenotypes, such as skin and/or heart diseases^13,35,39^, but only a few cases have been associated with esophageal dysfunction^21,40^. We associated *DSP* and *PPL* mutants with CAPN14, an esophageal epithelial specific protease involved in barrier integrity^8^. *DSP* or *PPL* missense variants likely introduce pathologic vulnerability to CAPN14 proteolysis and subsequent *DSP* or *PPL* insufficiency. Our data substantiated that EoE-associated variants had enhanced susceptibility to degradation by CAPN14. CAPN14 belongs to the classical subfamily of the calpain family on the basis of a similar domain structure^28^. Our findings mechanistically link CAPN14 with *DSP* and *PPL* insufficiency; notably, *DSP* and *PPL* are markedly reduced in the esophagus of patients with familial and non-familial EoE (Fig. 2a). We observed strong penetrance for most of these variants across multiple families, not only leading to morphologic change but also leading to functional change, as shown by the observed in vitro barrier impairment. Collectively, these findings provide a possible explanation for why the desmosomal variants found in our study are restricted to the esophagus, whereas variants shown by others lead to a variety of cardiocutaneous conditions, consistent with recent findings that post-transcriptional processes contribute to tissue-specific diseases^41^. Perhaps gene-environmental interaction, gene–gene interaction, and/or epigenetic regulation could be additional regulatory mechanisms controlling disease onset.

The pathologic vulnerability of the *DSP* and *PPL* variants to CAPN14 suggests possible avenues for therapeutic intervention. We indeed found that administering the calpain inhibitor SNJ-1945 in vitro ameliorates the CAPN14-mediated desmosomal degradation, at least in part. As CAPN14-mediated degradation is likely a common EoE mechanism not limited to mutations in *DSP* and *PPL*, these findings may substantiate the potential therapeutic value of calpain inhibitors in modifying the identified genetic pathway. Among the wide variety of calpain inhibitors^29^, we tested SNJ-1945, specifically designed to have increased water solubility and bioavailability due to its suitability for oral administration.

In conclusion, our study mechanistically substantiates a pathogenic role for barrier and desmosomal dysfunction in EoE and the likely intersection of this dysfunction with tissue-specific (calpain-14) and RhoGTPase–mediated pathways common to familial and non-familial EoE, suggesting a new approach for therapeutic intervention. We demonstrated that a series of rare genetic variants in the genes encoding the desmosome-associated proteins *DSP* and *PPL* contributes to EoE in 21% of patients with familial EoE, likely by impairing epithelial barrier function. Furthermore, we present evidence that loss of *DSP* and *PPL* occurs in non-familial EoE, providing a mechanism common for familial and non-familial EoE. We show that non-familial EoE has an acquired dysregulation (differential *DSP* and *PPL* expression on the basis of disease activity), whereas the defect in multiplex families is fixed genetically, as demonstrated by the retained dilated intercellular spaces (DIS) and decreased expression of *DSP* and *PPL* even during disease remission. These differences corroborate the established importance of environmental and genetic factors^1,4^, the variation in EoE observed in clinical practice and the predisposition of EoE to recur upon cessation of current treatments. Our findings mechanistically link *DSP* and *PPL* variants with increased susceptibility to CAPN14-mediated degradation, providing a possible explanation for the tissue-specific nature of familial EoE. Additionally, we demonstrate the potential loss of RhoGTPase activity as a consequence of these variants and that this process may contribute to EoE even in patients without primary genetic variants in *DSP* and *PPL*. These findings underscore a pathogenic role for desmosomal dysfunction in EoE and the likely intersection of this with CAPN14– and RhoGTPase–mediated pathways. As therapeutic mainstays for EoE currently target inflammation (e.g., corticosteroids) or indicate allergen avoidance (e.g., dietary therapy), these findings prompt the development of therapeutics to normalize epithelial barrier function focused on desmosome function and provide a means for improved diagnostics based on targeted genome sequencing. These findings also highlight the value of WES in deciphering complex traits, providing a paradigm for assessing complex traits by starting with familial cases and extending the findings to the more common presentation.

## Methods

### Study participants and design

We recruited patients with eosinophilic esophagitis (EoE) and family members in Cincinnati Children’s Hospital Medical Center (CCHMC), Cincinnati, USA, from 2005 through 2017. Diagnosis of EoE was defined as symptoms consistent with esophageal inflammation and/or dysfunction and ≥15 eosinophils per high-power field (eos/hpf) in distal esophageal biopsies regardless of concurrent proton pump inhibitor (PPI) use, consistent with international consensus diagnostic criteria^42^. Subjects with histologically confirmed EoE were defined as “affected”, whereas all other phenotypes, including subjects with no histologic evidence of EoE, were defined as “unaffected”. All families were ascertained through a proband with EoE. In addition to familial EoE (62 multiplex families), controls and non-familial EoE (non-multiplex families) were also included in the study for histologic and molecular analysis.

Whole-exome sequencing (WES) was performed on 62 unrelated families with European ancestry who have multiple family members with EoE across the US (21 states). The cohort of patients comprised two sets: Family F430 (as discovery set) and 61 families (as replication set). The first step was to identify a variant that segregated with affected family members of a large pedigree with EoE (Family F430). We sequenced the exomes of five affected family members (Patients 1, 2, 4, 11, and 19). After observing the presence of a variant in each of the five affected members, we then performed Sanger sequencing of samples obtained from all 32 Family F430 members, including spouses, to determine whether the same variant co-segregated with affected status. The second step involved screening for additional variants of the candidate EoE genes in the 61 families with EoE. We sequenced the exome of one affected family member (index patient) per family, followed by Sanger sequencing for all first-degree relatives of the index patients. The third step was, if a gene were identified as a strong candidate, to obtain evidence for its role in disease pathogenesis using functional biologic assays. The identification of variants that were functionally shown to be pathogenic in a gene known to encode a protein that has a critical role in epithelial homeostasis would provide evidence of its function in EoE pathogenesis.

This study involving human subjects was conducted under the approved IRB (Cincinnati Children’s Hospital Medical Center) protocol number 2008-0090. An informed consent/assent form including a consent to publish an indirect identifier was received from the subjects and/or their legal guardians per institutional guidelines prior to inclusion in the study. Study participants were also made aware that their involvement in the study was voluntary, and their declination to participate did not interfere with their standard of care.

### Whole-exome sequencing (WES)

Genomic DNA was extracted with the use of kits that were designed to obtain saliva samples (OG-500, DNA Genotek) or peripheral blood (QIAamp DNA Blood Mini Kit, Qiagen, Hilden, Germany), according to the manufacturer’s instructions. WES was performed in the CCHMC Genetic Variation and Gene Discovery Core, the Oklahoma Medical Research Foundation (OMRF) Genomics facility, Perkin Elmer, and Broad Institute. Libraries were prepared using the Illumina TruSeq capture kit and sequenced on an Illumina HiSeq2000 or HiSeq4000 to generate 100–150-base, paired-end reads. Sequencing reads were aligned using Burrows–Wheeler Aligner (BWA) and GRCh37 human reference genome, and variant calls were made simultaneously following the Genome Analysis Toolkit (GATK) Best Practices by using the GATK Unified Genotyper^43–45^.

### WES variant filtering

Variant filtering was performed using SNP and Variation Suite (SVS) software (Golden Helix Inc.) and the Cincinnati Analytical Suite for Sequencing Informatics (CASSI)^46^. Filters included minimum read depths >15 and genotype quality scores >20. Genotype calls were filtered using alternate allele ratios of <0.15 for homozygotes for the reference allele, 0.30–0.70 for heterozygotes, and >0.85 for homozygotes for the alternate allele.

### Sanger sequencing

All variants identified by WES were validated by Sanger sequencing of PCR amplified DNA. Of all families with the proband having variants, Sanger sequencing was performed on all affected and unaffected members whose DNA samples were available. Primer pairs were designed with the software program (primer 3) to amplify 100–300 bp from the variants. The primers are reported in Supplementary Table 10. PCR was performed using 50 ng DNA, dNTPs (200 μM), DMSO (3%), forward and reverse primers (0.5 μM, each) and 0.2 μL Phusion DNA polymerase (New England Biolabs) in 20-μL reaction volumes. Samples were amplified using Proflex PCR System (Applied Biosystems) with a program of 98 °C for 30 s; 35 cycles of 98 °C for 10 s, annealing temperature 55 °C to 70 °C for 30 s and 72 °C for 30 s; and 72 °C for 7 min at the end. Cycle sequencing was performed on the purified products at Genewiz (South Plainfield). Chromatograms were analyzed using SnapGene software (GSL Biotech).

For Family F430 (as a discovery set), we examined for Mendelian inheritance and co-segregation of variants to evaluate autosomal dominant inheritance.

To evaluate the generalizability of the *DSP* and *PPL* findings, we examined the unrelated 61 probands with exome data and identified an additional 12 families in which the proband had *DSP* and/or *PPL* variants. We then evaluated the likelihood of co-occurrence of the variant with the phenotype among affected family members by scoring the number of relatives of probands whose phenotype predicted genotype (presence or absence of variant). The likelihood of each relative sharing the coding variant with proband is independent of other relatives; thus, each relative is considered as an independent event. For parents, who each contribute genetic information to the proband, the genotype of one parent can be used to infer the genotype of the other parent, so parent dyads were considered. To evaluate whether the results would be predicted by chance, we assumed that 50% of first-degree relatives (# siblings + # of parent dyads) would have discordance between phenotype and genotype (e.g., unlinked). We then calculated the probability of the data given chance using the binomial distribution.

### Minor allele frequencies

Rare variants were annotated by Annotate Variation (ANNORVAR)^47^, identified and filtered using public data from the NCBI RefSeq genome build 105, the 1,000 Genomes Project (1 kG Phase1 - Variant Frequencies 2012_04_26 v3, GHI)^48^, the NHLBI GO Exome Sequencing Project (NHLBI ESP6500SI-V2 Exomes Variant Frequencies 0.0.19, GH)^49^ and the Exome Aggregation Consortium (ExAC) (ExAC Variant Frequencies 0.3 v2, BROAD)^14^. Rare variants were filtered using a minor allele frequency (MAF) cutoff of <0.001 (0.1%) in the European ancestry populations. In addition to the public databases, variant frequencies were filtered by using a MAF cutoff of <0.01 (1%) against an internal CCHMC database of WES data from 357 patients with various diseases, including macrophage-activating syndrome, systemic lupus erythematosus and juvenile idiopathic arthritis^46^.

### Algorithms predicting conservation and pathogenicity

Functional predictions were made using the database for nonsynonymous single-nucleotide variant functional predictions (dbNSFP)^50–52^. The theoretical pathogenicity of variants was assessed by applying algorithms (SIFT, PolyPhen2-HDIV and HVAR, LRT, Mutation Taster, Mutation Assessor, FATHMM, CADD, Radial SVM, and LR)^53–60^ that calculate amino acid conservation and the likelihood of deleteriously altering the encoded amino acid function. Variants were considered to be potentially damaging if they were predicted to be “Damaging”, “Possibly damaging”, “Deleterious” or “Functional” in at least 1 of these algorithms (SIFT, PolyPhen2-HDIV and HVAR, LRT, Mutation Taster, Mutation Assessor, FATHMM, CADD, Radial SVM, and LR) on the basis of thresholds by previous reports^60,61^. To assess whether variants are highly conserved across multiple species, we used the Multiz alignment of 62 mammalian species available on the UCSC Genome Browser^62^ to determine whether the amino acid substitution would compromise function. A variant allele that is conserved across species would be assumed to lack variation because such variations and changes would carry a deleterious effect on the organism. Algorithms (PhyloP, SiPhy, and GERP + + )^63–65^ also included information concerning known evolutionary conservation at the level of DNA base pairs. Deleteriousness prediction methods and thresholds were summarized in Table S5.

### Case-control association analysis

We examined whether the association of variants with EoE versus the association of variants with ancestry/race matched controls from the ExAC database^14^ reflected a statistically significant difference. We calculated the odds ratio (OR) using the chi-square test and statistical significance (*P*-values) using the Fisher’s exact test. For population control allele counts, we used race-matched population groups from the ExAC database^14^. Differences were considered as significant when *P*-values were <0.05.

### Rare variant burden analysis

To evaluate whether rare variants in specific genes were enriched in the case cohort compared to controls, association testing was performed by the Fisher’s exact test. Rare variants were filtered using a MAF cutoff of <0.001 (0.1%) in the European ancestry populations from the ExAC database^14^. Cases included the 62 unrelated index patients with EoE. Controls included 33,370 control samples from the ExAC database^14^ and 4,822 non-EoE control samples from the UK Biobank^15^, respectively. Given the potential population stratification, only European ancestry was included in the analyses. Desmosomal genes were defined by gene ontology term (GO:0030057, 25 genes).

### SNP genotyping

DNA from patients was obtained from saliva or peripheral blood samples. rs10192210 in *CAPN14* was genotyped in 62 unrelated families with European ancestry who have multiple family members with EoE by using TaqMan assay [CAPN14 (rs10192210), C___8374570_20]^66^.

### Clinical characteristics and symptom questionnaire

General clinical characteristics were gathered via research interviews at enrollment and at the time of endoscopy. Information on atopic history (asthma, allergic rhinitis, atopic dermatitis/eczema, and food allergy/anaphylaxis) was also collected. During research interviews at the time of endoscopy, we captured general clinical information relevant to EoE. Dichotomous yes/no answers regarding gastrointestinal symptoms, including dysphagia (food impaction and difficulty swallowing), gastroesophageal reflux disease (GERD; heartburn and reflux), nausea/vomiting (nausea and emesis), and pain (chest pain and abdominal pain), were gathered from patients or their guardians. Endoscopic features were prospectively recorded in real-time using a classification and grading system by a simplified endoscopic severity score (ESS) with each feature (edema, rings, exudates, furrows, and strictures) scored as absent or present. The endoscopic phenotype was based on the results of the ESS^67^. An inflammatory endoscopic phenotype was defined by findings limited to furrows, exudates, or edema, whereas a fibrostenotic endoscopic phenotype was defined if there were findings of rings or esophageal stricture. A normal endoscopic phenotype had no abnormalities visualized on endoscopy.

### Data management

All clinical data were collected on a paper form and entered into one of two electronic data sets^68^. Basic research data were collected through the use of a Structured Query Language Server database developed and maintained in the Cincinnati Center for Eosinophilic Disorders (CCED) at CCHMC. These data, as were all clinical laboratory data, were captured with DocFlowSheets in an Epic electronic medical record and were later extracted for analysis. Once the forms were all entered, the electronic record was compared with the paper forms to ensure that there were no discrepancies from data entry. Data extracted from the separate databases were then joined by using statistical software.

### Molecular expression and histologic analysis by esophageal biopsies

Esophageal biopsy specimens taken from familial EoE (62 multiplex families), 93 controls, and non-familial EoE (511 non-multiplex families) were analyzed using several approaches, including EoE Diagnostic Panel (EDP)^17^, single-cell RNA sequencing^18^, quantification of peak esophageal eosinophils, EoE Histology Scoring System (EoEHSS)^16^ and expression of *DSP* and *PPL* by western blot and immunofluorescence staining^69^. For non-familial EoE, children and adults with EoE but not from multiplex families and who were presenting for standard of care were independently enrolled in the study for histologic and molecular analyses. Active EoE was defined as esophageal biopsy specimens that showed 15 or more eosinophils/hpf, and inactive EoE was defined as less than 15 eosinophils/hpf in patients with a previous history of EoE^67^. Control subjects defined by the distal esophagus at biopsy having ≤2 eosinophils/hpf with no history of EoE despite presenting with symptoms typical of EoE. Though the condition of these patients may not be completely normal, they serve as a relevant control group for comparison with EoE^70^. Clinical characteristics for each analysis were summarized in Table S8.

### Endoscopic sample collection from patients

Subjects undergoing diagnostic endoscopy for ongoing clinical care consented/assented to provide additional esophageal biopsy specimens for research in addition to the standard clinical practice of obtaining esophageal endoscopic biopsy specimens. The research biopsy specimens were obtained after specimens had been obtained for clinical purposes. Distal esophageal biopsy was used throughout the study because this is typically obtained during endoscopy, represents the conventional location of biopsies, and allows ready comparison to the previous transcriptomic studies^17,67^, which are generally limited to this region. The tissues obtained for research were then placed in RNAlater buffer (Qiagen) and later processed for RNA extraction.

### PCR amplification of *DSP*, *PPL*, and representative EoE gene analysis

Fresh biopsy specimens were stored in RNAlater until they were subjected to RNA isolation using the miRNeasy kit (Qiagen) per the manufacturer’s instructions. Esophageal biopsy RNA (*N* = 246; 147 active EoE, 51 inactive EoE, and 48 unaffected controls) were isolated (Table S8 in the Supplementary information). After RNA quantity and quality analyses with a NanoDrop spectrometer, an aliquot of 500 ng of RNA was acquired for reverse transcription by using the iScript cDNA Synthesis Kit (Bio-Rad Laboratories), according to the manufacturer’s protocol.

In addition to *DSP* and *PPL* transcripts, the transcriptomic signature of esophageal biopsy samples was obtained using an EoE Diagnostic Panel (EDP) comprising a set of 96 esophageal transcripts (which includes housekeeping genes)^17,67^. *DSP* (Hs00189422_m1), *PPL* (Hs00160312_m1) and representative EoE genes in EDP were amplified from cDNA stock generated by the methods described above. The primers are reported in Supplementary Table 11. Using TaqMan Universal Master Mix II (Applied Biosystems), TaqMan real-time PCR amplification was performed on the Quant Studio 7 (Life Technologies). After the qPCR was complete, raw cycle threshold (CT) values for each sample/each gene were exported into GeneSpring GX 12.6 (Agilent Technologies) for statistical analysis. Glyceraldehyde 3-phosphate dehydrogenase (*GAPDH*) was used as an expression control for all analyzed genes. Samples with a *GAPDH* value of <30 CT value were considered acceptable for analysis. The expression CT value of the housekeeping gene *GAPDH* was subtracted from each gene of interest (GOI) CT value to acquire the ΔCT calculated^17,67^.

### Single-cell RNA sequencing

Single-cell suspensions were prepared from biopsies. Bulk population cells were directly subjected to the 10X mass genomics chip (10X Genomics, Inc.) targeting 10,000 simultaneously captured live events for next-generation sequencing. Each cell was uniquely barcoded during the cDNA library generation; libraries were subsequently sequenced on an Illumina HiSeq 2500 at CCHMC’s Genetic Variation and Gene Discovery Core, which allocated to a total read of ~320 million (two lanes/flow cell). Sparse data matrices, provided by 10X Genomics, were used as input into Seurat for further analysis^71^. For analysis of all sequenced esophageal samples, cells were filtered on the basis of unique feature counts >4800 or <200. Cells with >20% mitochondrial counts were filtered. Only genes expressed in at least three cells were retained. The total number of cells passing the filters and captured across all patients was 39,562 cells, and 20,208 genes passed the quality control check. Of these cells, 30,967 cells were classified as epithelial cells. Principal component analysis (PCA) was performed over the list of the variable genes. Data were subjected to Uniform Manifold Approximation and Projection (UMAP) and shared nearest neighbor (SNN) modularity optimization-based clustering. Using PCA and SNN modularity optimization-based clustering algorithm with a resolution of 0.5 and UMAP, we identified eight distinct clusters, including three epithelial clusters. Top marker genes with high specificity were used to classify cell markers into cell types on the basis of existing biological knowledge. To calculate relative expression, data were processed by the first log normalizing the expression and then scaling the data so that it was centered at zero.

### Histologic features

Histologic features were assessed by peak eosinophil counts and the EoE histology scoring system (HSS)^16^. Eight features of esophageal biopsies were defined and evaluated. Eosinophil inflammation (EI) was graded using peak eosinophil counts obtained by counting eosinophils in the most densely inflamed hpf. Additional features were basal zone hyperplasia (BZH), > 15% of the total epithelial thickness; eosinophil abscess (EA), solid mass of intraepithelial eosinophils; eosinophil surface layering (SL), linear alignment of eosinophils parallel to the epithelial surface; dilated intercellular spaces (DIS), spaces around squamous epithelial cells that exhibit intercellular bridges; surface epithelial alteration (SEA), surface epithelial cells that exhibit altered tinctorial properties, manifesting as dark red staining, with or without intraepithelial eosinophils; dyskeratotic epithelial cells (DEC), individual cells with deeply eosinophilic cytoplasm and hyperchromatic nuclei; and lamina propria fibrosis (LPF), thickened connective tissue fibers in the lamina propria. Each feature was scored separately for grade (severity) or stage (extent) of the abnormality using a 4-point scale (0 = normal; 3 = most severe or extensive). The total score for each feature was defined as the sum of the scores for grade and stage. Therefore, the total score for each feature ranges from 0–6 because each score ranges from 0–3. HSS features were also grouped into those that relate directly to eosinophilic inflammation (i.e., a score of peak eosinophil count, eosinophil abscesses, eosinophil surface layering, and surface epithelial alteration) and those that relate to architectural aspects (basal zone hyperplasia, dilated intercellular spaces, dyskeratotic epithelial cells, and lamina propria fibrosis). For each group, the ratio of the sum of the assigned total scores for each evaluated feature divided by the maximum possible score for that biopsy (range, 0–1).

### Immunofluorescence

For immunofluorescence of biopsies, *DSP* (sc-390975, Santa Cruz) (1:50 dilution) and *PPL* (ab131269, Abcam) (1:100 dilution) antibodies were used on at least 4 distal esophageal biopsies from control individuals and from patients with EoE with or without variants. Isotype antibodies were used as negative controls. The nuclei were stained with DAPI. The slides were blocked with PBS with 10% donkey serum. The secondary antibodies (1:400 dilution) used were donkey anti-mouse Alexa Fluor 488 or donkey anti-rabbit Alexa Fluor 647 (Invitrogen). Imaging was performed with a Nikon A1 inverted confocal microscope (Nikon). The fluorescence intensity of each biopsy was calculated using the ImageJ calculation for corrected total fluorescence = integrated density−(selected area of biopsy × mean fluorescence of background readings)^72^. For each gene, fluorescence intensity was determined with a ratio of control individuals to patients with EoE with or without variants for ease of interpretation.

### Cell culture

Various cell types were used in the study: EPC2 (hTERT-immortalized human esophageal epithelial cell line), HaCaT (immortalized human skin keratinocyte line), and HEK293T (human embryonic kidney cell line 293T). The EPC2 was a gift from Anil Rustgi (University of Pennsylvania, Philadelphia, Pennsylvania, USA) and was cultured in a keratinocyte serum-free medium (KSFM). Briefly, EPC2 cells, according to experimental conditions, were grown to confluence on semipermeable membranes. Cells were then grown submerged in high-calcium (1.8 mM) media for 5 days, after which the media from the upper chamber was removed; cells were then grown for an additional 6 days at the air–liquid interface (ALI). The HaCaT cell line was cultured in KSFM. HEK293T cells were cultured in Dulbecco’s Modified Eagle Medium (DMEM; Lonza) supplemented with 10% FBS.

### Generation of *DSP* or *PPL* gene-deficient EPC2 cells using CRISPR/Cas9-mediated mutagenesis

A guide RNA (gRNA) complementary to the (a) *DSP* or (b) *PPL* open reading frame sequence and located directly 5' of a protospacer adjacent motif (PAM) was identified for:

- *DSP*, 5'-CTGCGCTACGAGGTGACCAG-3' (http://www.broadinstitute.org/rnai/public/analysis-tools/sgrna-design)^73^
- *PPL*, 5'-GCTGCAGAAGAATGCCGACC-3' (http://crispor.tefor.net)^74^

The following oligonucleotides were annealed and ligated into the BbsI restriction site of plasmid pX459M2 (obtained from CCHMC Transgenic Mouse and Gene Editing Core Facility) to produce (a) pX459M2-DSPg2 and (b) pX459M2-PPLg1:

- *DSP* 5'-CACCGCGCTACGAGGTGACCAG-3' 5'-CGCGATGCTCCACTGGTCCAAA-3'
- *PPL* 5'-CACCGCTGCAGAAGAATGCCGACC-3' 5'-AAACGGTCGGCATTCTTCTGCAGC-3'

EPC2 cells were transduced with pX459M2, pX459M2-DSPg2, or pX459M2-PPLg1 using Viromer Red reagent (Origene) according to the manufacturer’s protocol. Transduced cells were selected using puromycin, expanded, dispersed, and plated by limiting dilution in 96-well plates in the presence of irradiated NIH 3T3 feeder cells. The resulting EPC2 cell clones were expanded, and genomic DNA was extracted using the Quick gDNA micro prep kit (Zymo Research) according to the manufacturer’s protocol. For each clone, genomic DNA in the vicinity of the predicted edited region was amplified with the following primers (a) *DSP*, using nested PCR, we first amplified the gDNA with RB8648 (5'-CAACACCAACACCCAGCTC-3') and RB8653 (5'-GTACGACCGAGTCCCTGTTC-3'), and then gel extracted the 662-bp product and amplified with RB8652 (5'-CCGACATGAGCTGCAACG-3') and RB8651 (5'-AAGTTCTTTCGGGACCTGGG-3'); and (b) *PPL*, we amplified with RB8710 (5'-GGAACAGCCCAACTACCTCA-3') and RB8711 (5'-CCAGGTTCCTCTCACAGAGC-3'). The PCR products were sequenced with (a) RB8652 or (b) RB8711 to assess whether editing to produce a frameshift occurred. Knockout of protein expression was confirmed by western blot analyses.

### *DSP* and *PPL* overexpression

For overexpression in EPC2 cells, the wild-type and identified mutants of *DSP* or *PPL* were synthesized into a pLVX-IRES-puro lentiviral vector by the In-Fusion method (Takara). The EPC2 cells were grown and transduced in KSFM. The viruses, obtained from a 60 mm dish of HEK293T cells, were precipitated using Lenti-X concentrator solution and resuspended in 300 μl of KSFM. KSFM (150 μl) was mixed with polybrene to a final concentration of 5 μg/ml and used to transduce EPC2 cells grown to 60–70% confluency on a 6-well plate by centrifugation at 2000 g at room temperature for 1 h. All cells underwent puromycin selection (1 μg/ml) for 1–2 weeks and were later kept in puromycin at 0.5 μg/ml. The puromycin was removed 24 h prior to the beginning of the experiment. Expression levels were validated by quantitative PCR and western blot analyses.

### Transepithelial electrical resistance (TEER) and fluorescein isothiocyanate (FITC)-dextran flux measurements

EPC2 cells were subjected to the culture at the ALI on 12-well filter inserts^69^. TEER and 3- to 5-kDa FITC-dextran (Sigma-Aldrich) paracellular flux was measured using an EVOM2 (World Precision Instruments) and a fluorescence plate reader (BioTek), respectively.

### Wound healing assay

For the wound healing assay, EPC2 cells and HaCaT cells transduced with *DSP* or *PPL* constructs were simultaneously seeded on a Culture-Insert 4 Well in μ-Dish 35 mm, high ibiTreat (Ibidi USA), grown until confluency. The Culture-Insert 4 Well allows for wound healing assays with four cell-free gaps with a 0.5 mm gap width and one center area with a 1 mm diagonal distance. After removal of the insert, cells were washed three times, incubated at 37 °C and 5% CO_2_, and observed at 8 h or 12 h post wounding. Each wound was imaged at the time of wounding (0 h) and 8 h or 12 h post wounding using an EVOS inverted microscope (Thermo Fisher Scientific). The gap area was quantified using the ImageJ plugin MRI Wound Healing Tool (http://dev.mri.cnrs.fr/projects/imagej-macros/wiki/Wound_Healing_Tool). Percent change in wound area was defined as [100% × (W0-W8)/W0 (W0: wound width at 0 h; W8: wound width at 8 h)].

### Active RhoA assay

Active RhoA was measured using the RhoA G-LISA Activation Assay Kit, according to the manufacturer’s recommendations (Cytoskeleton). Briefly, cells were washed and lysed by the ice-cold lysis buffer with a protease inhibitor cocktail. As for the esophageal tissue biopsies, the frozen tissue samples were homogenized in ice-cold lysis buffer with a protease inhibitor cocktail. Lysates were clarified by a 1 min centrifugation, and supernatants were snap-frozen in liquid nitrogen. Total protein contents were assayed by bicinchoninic acid (BCA) (Thermo Fisher), and 1.0 mg/mL protein samples were loaded onto the pre-coated plates provided with the RhoA G-LISA kit. The plate was placed on an orbital microplate shaker at 400 rpm for 30 min at 4 °C and then incubated with anti-RhoA primary antibody (1:250), followed by a secondary antibody (1:62.5), on an orbital microplate shaker at 400 rpm for 45 min each at room temperature. The plate was then incubated with the HRP detection reagent at 37 °C for 15 min; absorbance was read at 490 nm using a microplate reader (BioTek). Total RhoA levels of each lysate were quantified by western blot.

### Calpain-14–mediated degradation assay

For in vitro calpain-14 degradation assays, HEK293T cells were co-transfected with *DSP* or *PPL* (non-variant or mutants) and CAPN14-FLAG or enzymatically inactive CAPN14-C101A-FLAG using TransIT-LT (Mirus Bio), as indicated. After 48 h, transfected cells were lysed without a protease inhibitor cocktail. To equalize each cell lysate, total protein concentrations were measured by BCA (Thermo Fisher). Cell lysates were incubated for 15 min at 37 °C in an equal volume of assay buffer (40 mM Tris HCl, 50 mM NaCl, 2 mM DTT) with and without 1 mM CaCl_2_. For calpain inhibitor assay, samples were pre-incubated with SNJ-1945 (250 μM)^75^ for 2 min at room temperature before addition of assay buffer. Reactions were terminated by the addition of 4 × protein sample buffer followed by boiling for 5 min. The resulting products were separated on SDS-PAGE gel and processed by western blot analysis. The protein remaining after activation of CAPN14 was determined by the difference in band intensity [e.g., (100 × after activation)/before activation]. For each mutation, total protein remaining was determined with a ratio of mutant cells to non-variant cells [Total protein (%) = each protein remaining/average of non-variant cells] for ease of interpretation.

### Western blot

Proteins from cell cultures were extracted with RIPA Lysis and Extraction Buffer (Thermo Fisher Scientific) with protease and phosphatase inhibitors unless otherwise noted. Loading buffer (Life Technologies) was added, and samples were heated to 95 °C for 5 min, subjected to electrophoresis on 4–12% NuPAGE Bis-Tris gels (Life Technologies), transferred to nitrocellulose membranes (Life Technologies), subjected to western blot analysis and visualized using the Odyssey CLx system (LI-COR Biosciences). The primary antibodies were anti-*DSP* (MAB9080, R&D Systems) (1:2000 dilution), anti-PPL (ab131269, Abcam) (1:2000 dilution) and anti-FLAG (F3165, Sigma-Aldrich) (1:2000 dilution). Anti-GAPDH antibodies (ab181602, Abcam; TA802519, Origene) (1:2000 dilution) and anti-HSP90 antibodies (ab13495, Abcam; TA500494, Origene) (1:2000 dilution) were used as loading controls. The secondary antibodies used were IRDye 680RD goat anti-mouse (LI-COR Biosciences) (1:10,000 dilution) and IRDye 800CW goat anti-rabbit (LI-COR Biosciences) (1:10,000 dilution). Blots were quantified using the Image Studio software (LI-COR Biosciences).

### Statistical analysis

Statistical analyses were performed using the JMP v13.1 (SAS Institute, Cary, NC), R statistical computing environment (version 3.1.2), GeneSpring GX 12.6 (Agilent Technologies, Santa Clara, CA) and GraphPad Prism 8 (GraphPad Software, Inc., San Diego, CA). Descriptive studies were not tested for statistical significance. Data are presented as n (%) or mean ± standard error of the mean (SEM) unless otherwise stated. Missing data were excluded from all statistical analyses. For categorical data, the chi-square test or Fisher’s exact test was used to ascertain differences. For continuous data, some variables exhibited normal distributions, whereas others exhibited deviations; thus, we employed both parametric and non-parametric tests to compare groups as appropriate. For the comparison of two groups, either *t*-tests or Mann–Whitney *U* test were used. For the comparison of three or more groups, we used one-way analysis of variance (ANOVA) followed by a Dunnett’s multiple-comparison test or the Kruskal–Wallis test followed by a Dunn multiple-comparison test. Correlations were calculated using Spearman correlations. Benjamini–Hochberg correction was applied for multiple testing. A significant *P*-value was defined as <0.05.

### Reporting summary

Further information on research design is available in the Nature Research Reporting Summary linked to this article.

## Supplementary information

Supplementary Information

Reporting Summary

## Data Availability

The patients’ genome data obtained by whole-exome sequencing during the current study are not fully available in a public or closed repository because study participants did not give full consent for releasing data; a subset of the sequences is deposited in the dbGAP accession phs001011.v2.p1. These data can be accessed under the condition that a joint research plan is made by the researchers and approved by the ethics committees. The esophageal molecular expression data by EDP and single-cell RNA sequencing have been deposited in EGIDExpress (https://egidexpress.research.cchmc.org/data/). All other data supporting the findings of this study are included within the article or the Supplementary information and are available from the corresponding author upon request. Source data are provided with this publication.

## Source data

Source Data
