# Supplementary material for: Desmoplakin and periplakin genetically and functionally contribute to eosinophilic esophagitis
Source: Nat Commun. 2021 Nov 23;12:6795. doi: 10.1038/s41467-021-26939-9 (PMC8611043; doi:10.1038/s41467-021-26939-9)
Supplement: Supplementary file 1 — Supplementary Information [file 41467_2021_26939_MOESM1_ESM.pdf]

Supplementary information

Desmoplakin and Periplakin Genetically and Functionally Contribute to Eosinophilic Esophagitis

Tetsuo Shoda, M.D., Ph.D.[1], Kenneth M. Kaufman, Ph.D.[2, 3, 4], Ting Wen, M.D., Ph.D.[1, 3], Julie M. Caldwell, Ph.D.[1], Garrett A. Osswald, B.S.[1], Pathre Purnima, B.S.[1], Nives Zimmermann, M.D.[1, 3, 5], Margaret H. Collins, M.D.[3, 5], Kira Rehn, B.S.[1], Heather Foote, B.S.[1], Michael D. Eby, B.S.[1], Wenying Zhang, M.D., Ph.D.[3, 6], Netali Ben-Baruch Morgenstern, Ph.D.[1], Adina Y. Ballaban, B.S.[1], Jeff E. Habel, Ph.D.[1], Leah C. Kottyan, Ph.D.[1, 2, 3], J. Pablo Abonia, M.D.[1, 3], Vincent A. Mukkada, M.D.[3, 7], Philip E. Putnam, M.D.[3, 7], Lisa J. Martin, Ph.D.[3, 6] and Marc E. Rothenberg, M.D., Ph.D.[1, 3]

[1]Division of Allergy and Immunology, Cincinnati Children's Hospital Medical Center, 3333 Burnet Ave, Cincinnati, OH, 45229, USA, [2]Center for Autoimmune Genomics and Etiology, Cincinnati Children's Hospital Medical Center, 3333 Burnet Ave, Cincinnati, OH, 45229, USA, [3]Department of Pediatrics, University of Cincinnati College of Medicine, 3200 Burnet Avenue, Cincinnati, OH, 45229, USA, [4]Department of Research, Cincinnati Veterans Affairs Medical Center, 3200 Vine St, Cincinnati, OH 45220, USA, [5]Division of Pathology, Cincinnati Children's Hospital Medical Center, 3333 Burnet Ave, Cincinnati, OH, 45229, USA, [6]Division of Human Genetics, Cincinnati Children's Hospital Medical Center, 3333 Burnet Ave, Cincinnati, OH, 45229, USA, [7]Division of Gastroenterology, Hepatology and Nutrition, Cincinnati Children's Hospital Medical Center, 3333 Burnet Ave, Cincinnati, OH, 45229, USA.

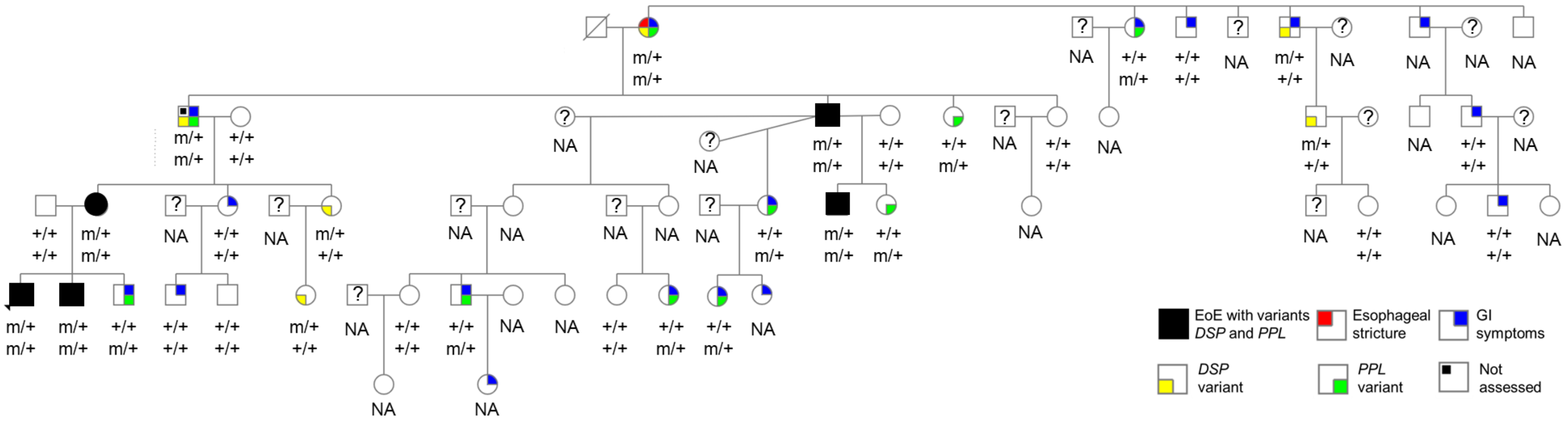

Supplementary Figure 1

Details in extended pedigree as discovery set.

Color symbols indicate phenotype. The arrowhead in the lower left indicates the proband, and the slash indicates a deceased subject. “Not assessed” indicates the subject having GI symptoms but had never undergone an esophagogastroduodenoscopy. In regard to variant genotyping, “m” indicates the mutant *DSP* or *PPL* allele and “+” the reference allele (*DSP* upper, *PPL* lower). Individuals with “?” have an unknown phenotype status and were not genotyped. EoE, eosinophilic esophagitis; GI, gastrointestinal; NA, not available.

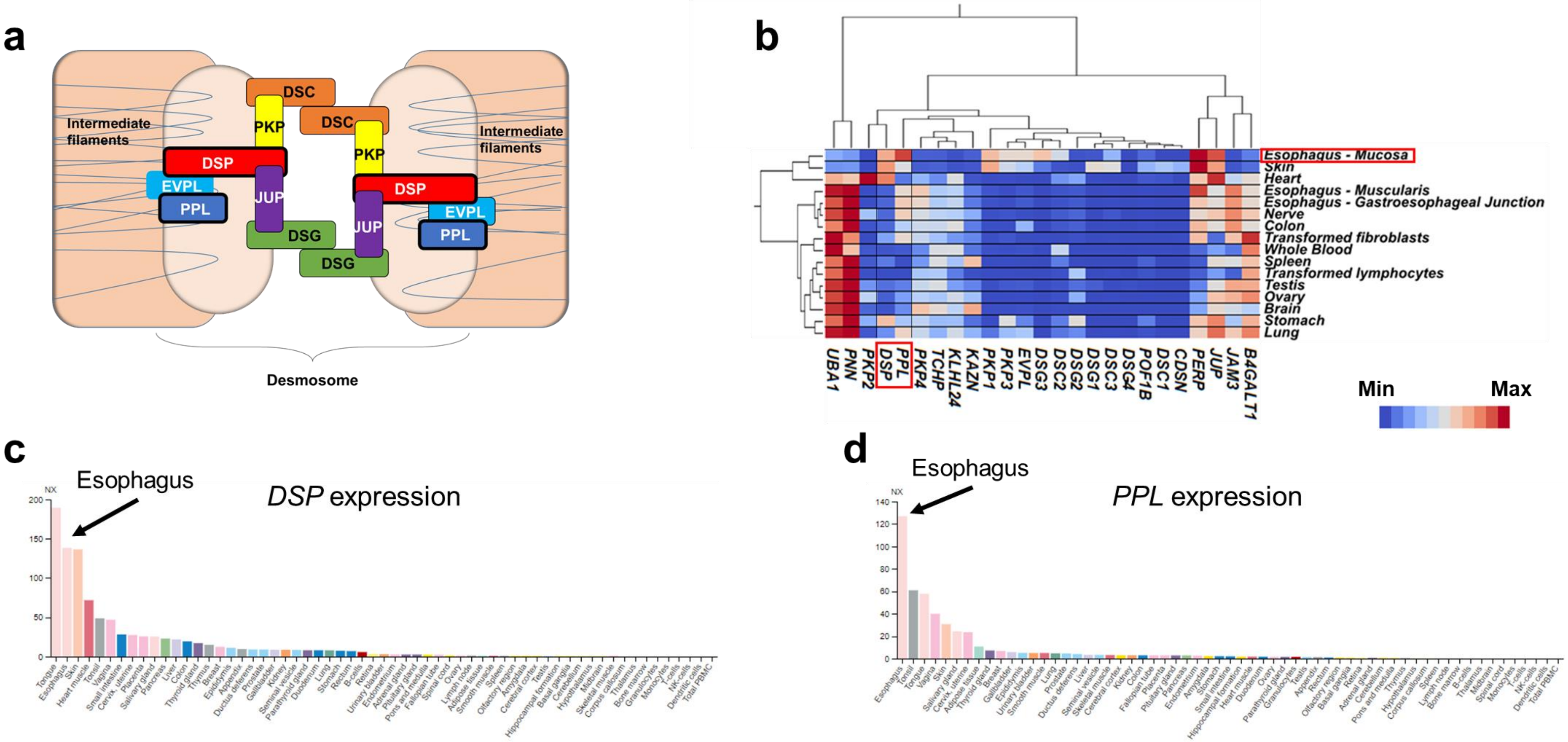

Supplementary Figure 2

Desmosome gene expression in normal tissues and cells.

a, Schema depicting the desmosomes. DSG, desmoglein; DSP, desmoplakin; EVPL, envoplakin; JUP, junction plakoglobin; PKP, plakophilin; PPL, periplakin. **b,** Expression heatmap of desmosomal genes across human tissues from the Genotype-Tissue Expression

(GTEx) database (https://wwwgtexportal.org/home/). **c–d,** *DSP* (c) and *PPL* (d) mRNA expression in normal human tissues from the Human Protein Atlas (HPA) database (https://www.proteinatlas.org/). The arrow indicates the esophagus.

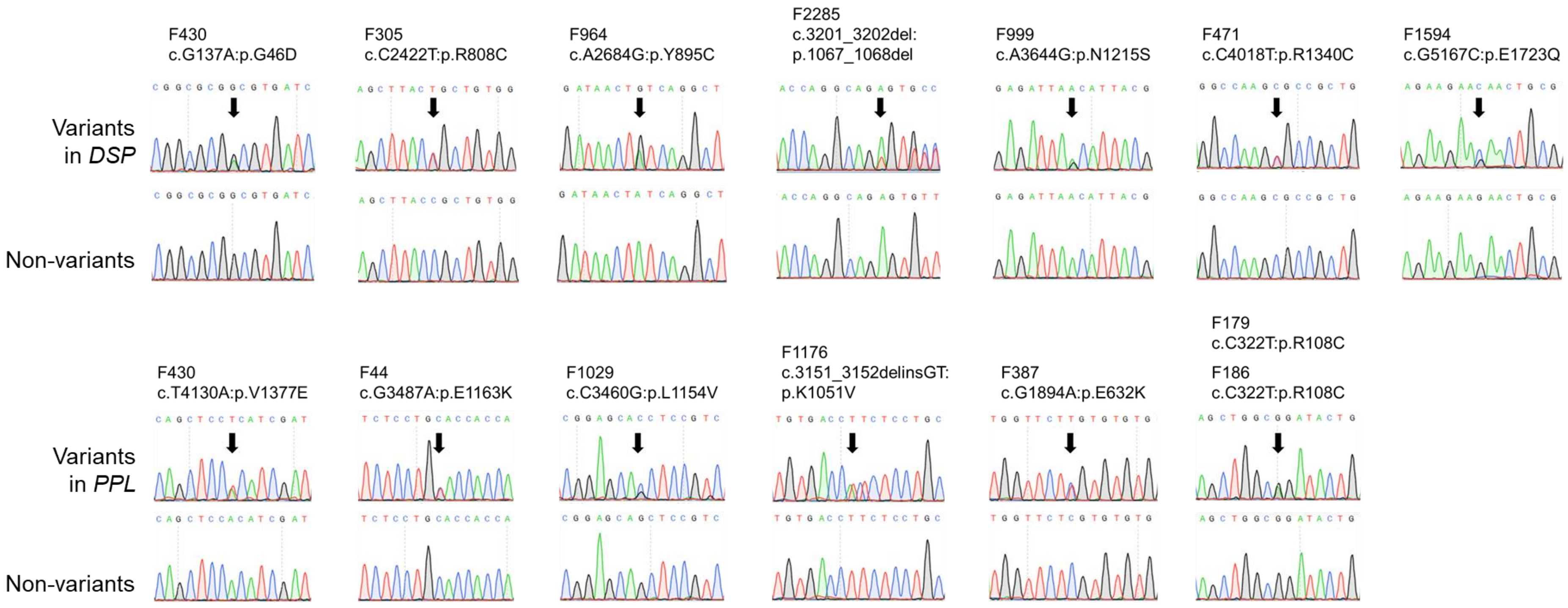

Supplementary Figure 3

Sanger sequencing results of variant compared to non-variant *DSP* and *PPL*.

For comparison, the corresponding non-variant sequences are shown below each mutated sequence in *DSP* and *PPL* identified in 13 of 62 multiplex families with eosinophilic esophagitis (EoE). The arrow indicates where the variants are in each patient.

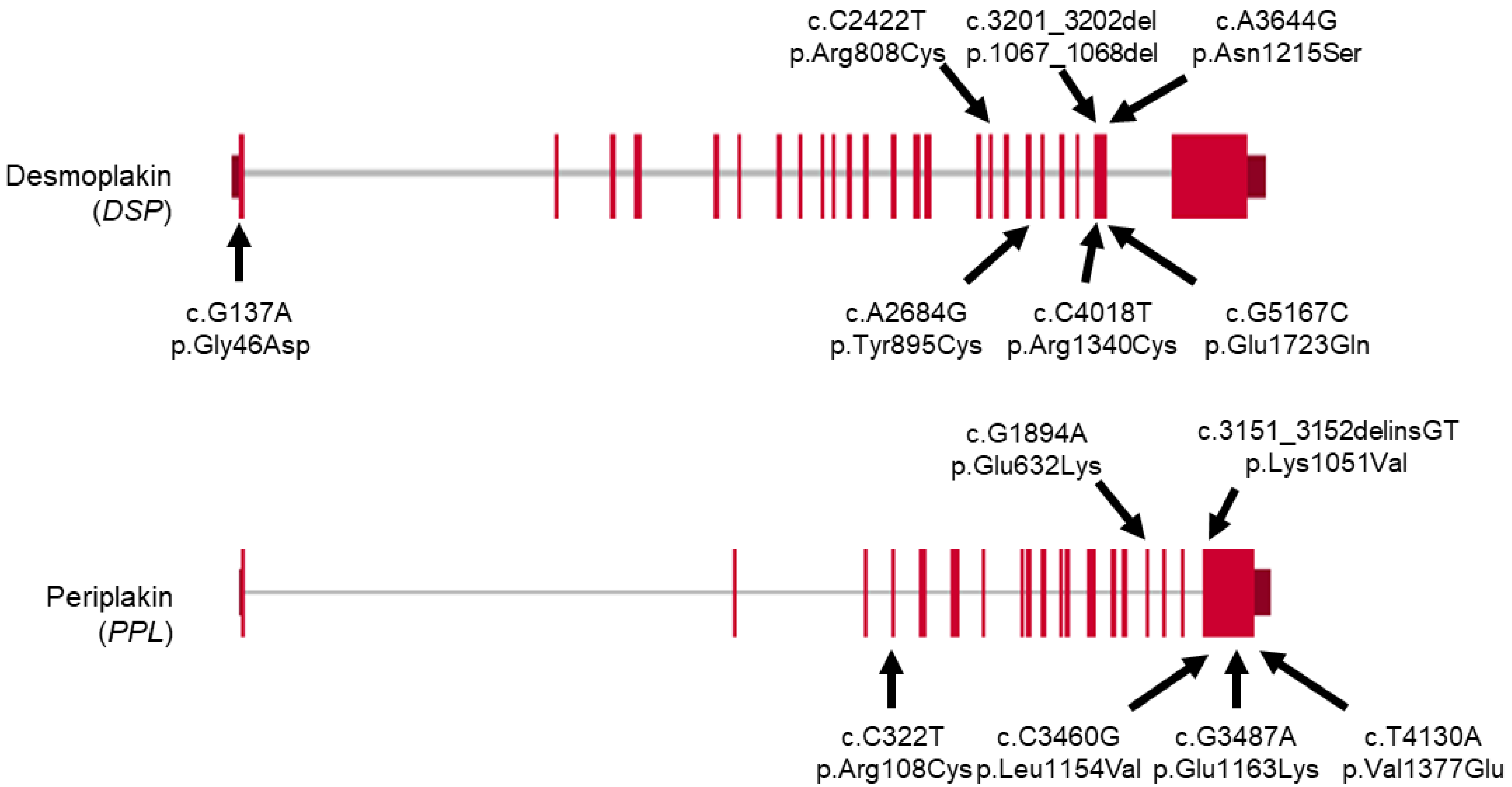

Supplementary Figure 4

Schematic of the *DSP* and *PPL* gene loci.

The *DSP* and *PPL* variants noted above the gene locus schematics are those identified by whole-exome sequencing (WES) in this study in 13 of 62 multiplex families with eosinophilic esophagitis (EoE) (discovery and replication sets).

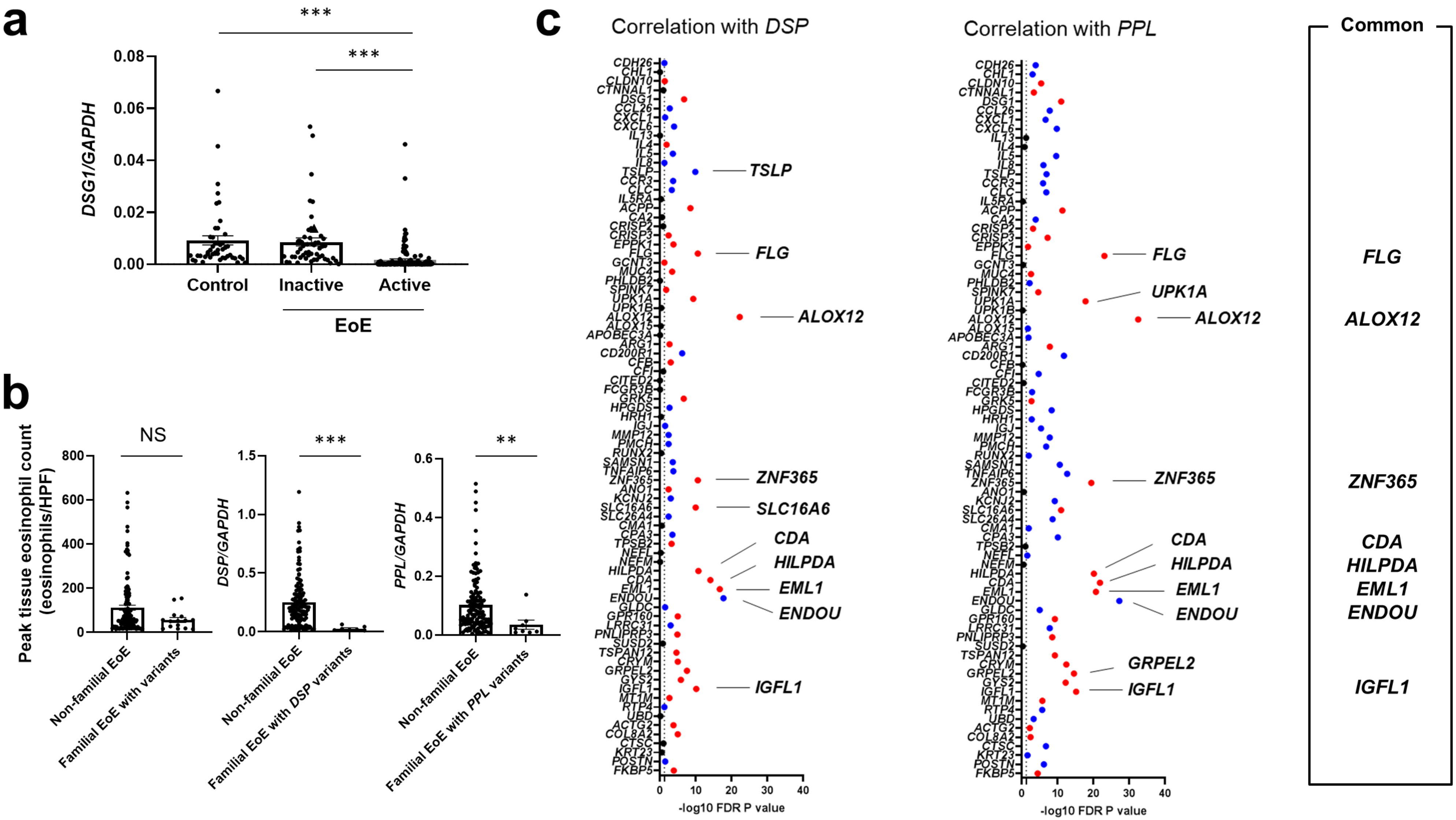
a
***
***
0.08
0.06
0.04
0.02
0.00
DSG1/GAPDH
Control
Inactive
Active
EoE
b
NS
800
600
400
200
0
Peak tissue eosinophil count (eosinophils/HPF)
Non-familial EoE
Familial EoE with variants
***
1.5
1.0
0.5
0.0
DSP/GAPDH
Non-familial EoE
Familial EoE with DSP variants
**
0.6
0.4
0.2
0.0
PPL/GAPDH
Non-familial EoE
Familial EoE with PPL variants
c
Correlation with DSP
TSLP
FLG
ALOX12
ZNF365
SLC16A6
CDA
HILPDA
EML1
ENDOU
IGFL1
-log10 FDR P value
Correlation with PPL
FLG
UPK1A
ALOX12
ZNF365
CDA
HILPDA
EML1
ENDOU
GRPEL2
IGFL1
-log10 FDR P value
Common
FLG
ALOX12
ZNF365
CDA
HILPDA
EML1
ENDOU
IGFL1

Supplementary Figure 5

Molecular expression analysis of esophageal biopsies.

a, *DSG1* mRNA expression from esophageal biopsies from controls and patients with eosinophilic esophagitis (EoE, inactive and active). Data points represent individual patients (Control, n = 48; Inactive EoE, n = 51; Active EoE, n = 147). Statistics: Control vs. Inactive EoE, >0.9999; Control vs. Active EoE, P < 0.0001; Inactive EoE vs. Active EoE, P < 0.0001. **b,** peak esophageal eosinophil count (left) and *DSP* (middle) or *PPL* (right) mRNA expression of patients with active EoE from non-familial EoE and familial EoE with variants (non-familial EoE, n = 115; familial EoE with *DSP* variants, n = 7; familial EoE with *PPL* variants, n = 8). Statistics: peak esophageal eosinophil count, P = 0.1374; *DSP*, P < 0.0001; *PPL*, P = 0.0028. **c,** Negative log10 FDR P-value of the Spearman correlation between *DSP* (left) or *PPL* (right) mRNA expression and a diagnostic subset of genes from the Eosinophilic Esophagitis Diagnostic Panel (EDP).[28] Red indicates a positive correlation, and blue indicates a negative correlation. The top 10 genes of EDP correlation are labeled, and the 8 common genes between *DSP* and *PPL* from their respective top 10 genes of EDP correlation are listed (far right). For **a-b**, data are presented as mean ± SEM. For **a-b**, n is the number of biologically independent subjects, and two-tailed P values were determined by the Kruskal-Wallis test followed by a Dunn multiple-comparison test (**a**) or by the Mann-Whitney *U* test (**b**). **P < 0.01 and ***P < 0.001. FDR, false discovery rate; NS, not significant; SEM, standard error of the mean.

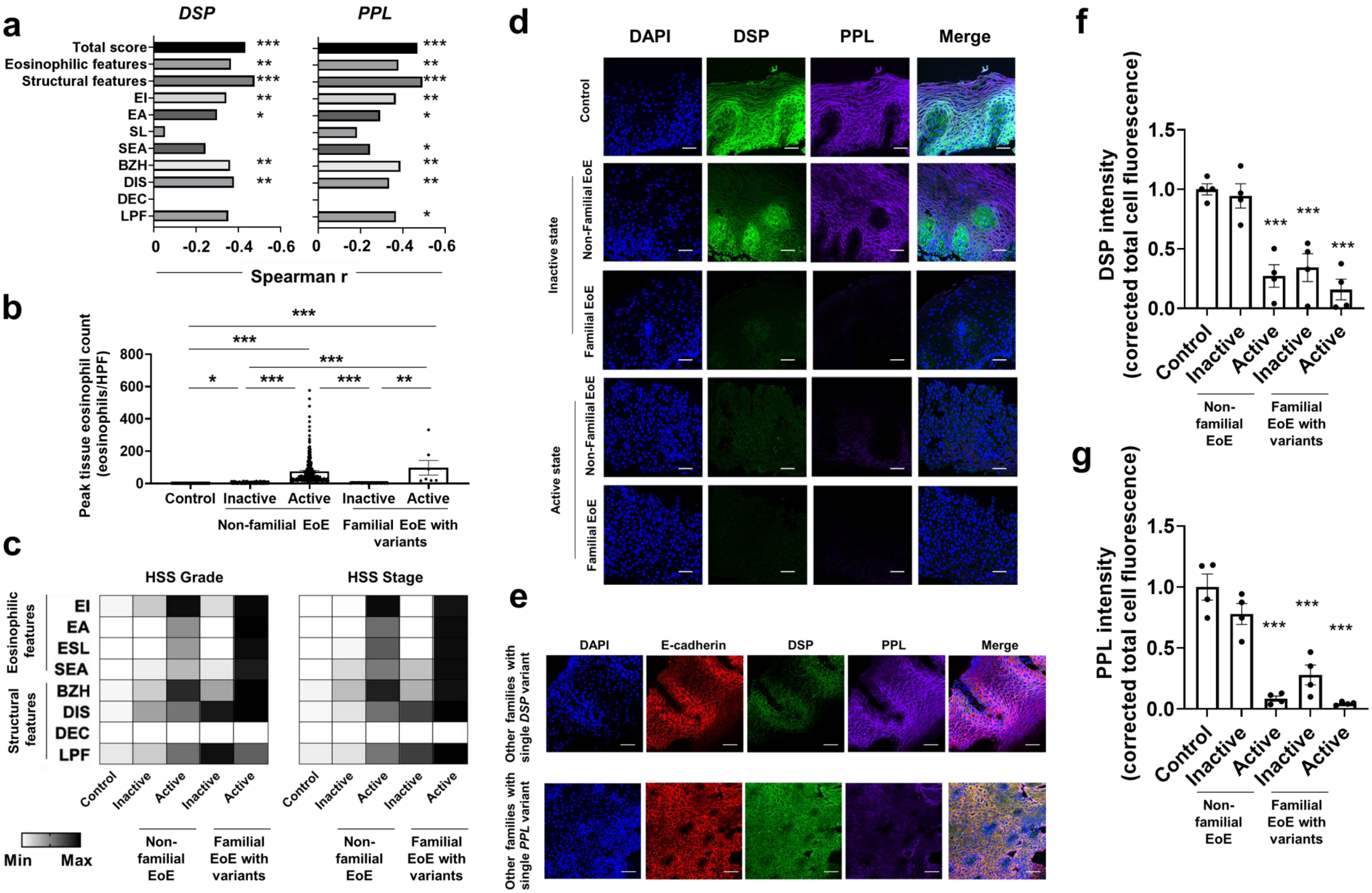

a
DSP
PPL
Total score
Eosinophilic features
Structural features
EI
EA
SL
SEA
BZH
DIS
DEC
LPF
Spearman r
b
Peak tissue eosinophil count (eosinophils/HPF)
Control
Inactive
Active
Non-familial EoE
Familial EoE with variants
c
HSS Grade
HSS Stage
Eosinophilic features
Structural features
EI
EA
ESL
SEA
BZH
DIS
DEC
LPF
Min
Max
d
DAPI
DSP
PPL
Merge
Control
Inactive state
Active state
Non-Familial EoE
Familial EoE
e
DAPI
E-cadherin
DSP
PPL
Merge
Other families with single DSP variant
Other families with single PPL variant
f
DSP intensity (corrected total cell fluorescence)
Non-familial EoE
Familial EoE with variants
g
PPL intensity (corrected total cell fluorescence)

Supplementary Figure 6

Histopathologic findings in patients with variants in *DSP* and *PPL*.

a, Correlations between *DSP* and *PPL* expression and Histology Scoring System (HSS) features. Spearman r values for correlations between the HSS composite scores and features (n = 68). **b,** Peak esophageal eosinophil count among control individuals, patients with non-familial EoE and patients with familial EoE with variants [Control, n = 58; non-familial EoE (Inactive, n = 254; Active, n = 271); familial EoE with variants (Inactive, n = 14; Active, n = 7)]. Statistics: Control vs. Inactive Non-familial EoE, P = 0.0263; Control vs. Active Non-familial EoE, P < 0.0001; Control vs. Inactive Familial EoE, P > 0.9999; Control vs. Active Familial EoE, P < 0.0001; Inactive Non-familial EoE vs. Active Non-familial EoE, P < 0.0001; Inactive Non-familial EoE vs. Inactive Familial EoE, P > 0.9999; Inactive Non-familial EoEvs. Active Familial EoE, P = 0.0002; Active Non-familial EoE vs. Inactive Familial EoE, P < 0.0001; Active Non-familial EoE vs. Active Familial EoE, P > 0.9999; Inactive Familial EoE vs. Active Familial EoE, P = 0.0011. **c,** Heat map demonstrating the average score of individual histologic features among control individuals, patients with non-familial EoE and patients with familial EoE with variants; more intense coloration indicates a greater degree of individual histologic features. Statistical differences in total scores of histologic features among these groups are listed in Table S9. **d–e,** Immunofluorescence staining of esophageal biopsy sections for DSP (cyan) and PPL (magenta) with DAPI–stained nuclei (blue). Data are representative of three experiments performed in duplicate. Scale bar: 100 µM; **d,** representative images of sections from normal control individuals, patients with non-familial EoE and a patient with EoE and *DSP* and *PPL* variants (individual 1 from family 430) during active and inactive (remission) disease state. **e,** representative images of sections from patients with EoE and DSP (individual 1 from family 1594) or PPL variants (individual 1 from family 1029) during inactive (remission) disease state with counterstain by E-cadherin (red) (less perturbed during the inactive EoE disease state). **f–g,** quantification of DSP (f) and PPL (g) fluorescence staining intensity. Data are representative of four experiments performed in duplicate. Statistics (versus Control): **f,** non-familial EoE (Inactive, P = 0.9792; Active, P = 0.0002); familial EoE with variants, (Inactive, P = 0.0006; Active, P < 0.0001); **g,** non-familial EoE (Inactive, P = 0.1392; Active, P < 0.0001); familial EoE with variants, (Inactive, P < 0.0001; Active, P < 0.0001). For **b, f** and **g**, data are presented as mean ± SEM. For **a-c** and **f-g**, two-tailed P values were determined by the following tests: **a** and **c**, Spearman's rank correlation coefficient (Benjamini–Hochberg correction was applied for multiple testing); **b**, Kruskal-Wallis test followed by a Dunn

multiple-comparison test; and **f-g**, one-way ANOVA test followed by a Dunnett's multiple-comparison test. *P < 0.05, **P < 0.01 and ***P < 0.001. DSP, desmoplakin; PPL, periplakin; FDR, false discovery rate; EI, eosinophilic inflammation; EA, eosinophilic abscess; ESL, eosinophilic surface layering; SEA, surface epithelial alteration; BZH, basal zone hyperplasia; DIS, dilated intercellular spaces; DEC, dyskeratotic epithelial cells; LPF, lamina propria fibrosis; SEM, standard error of the mean.

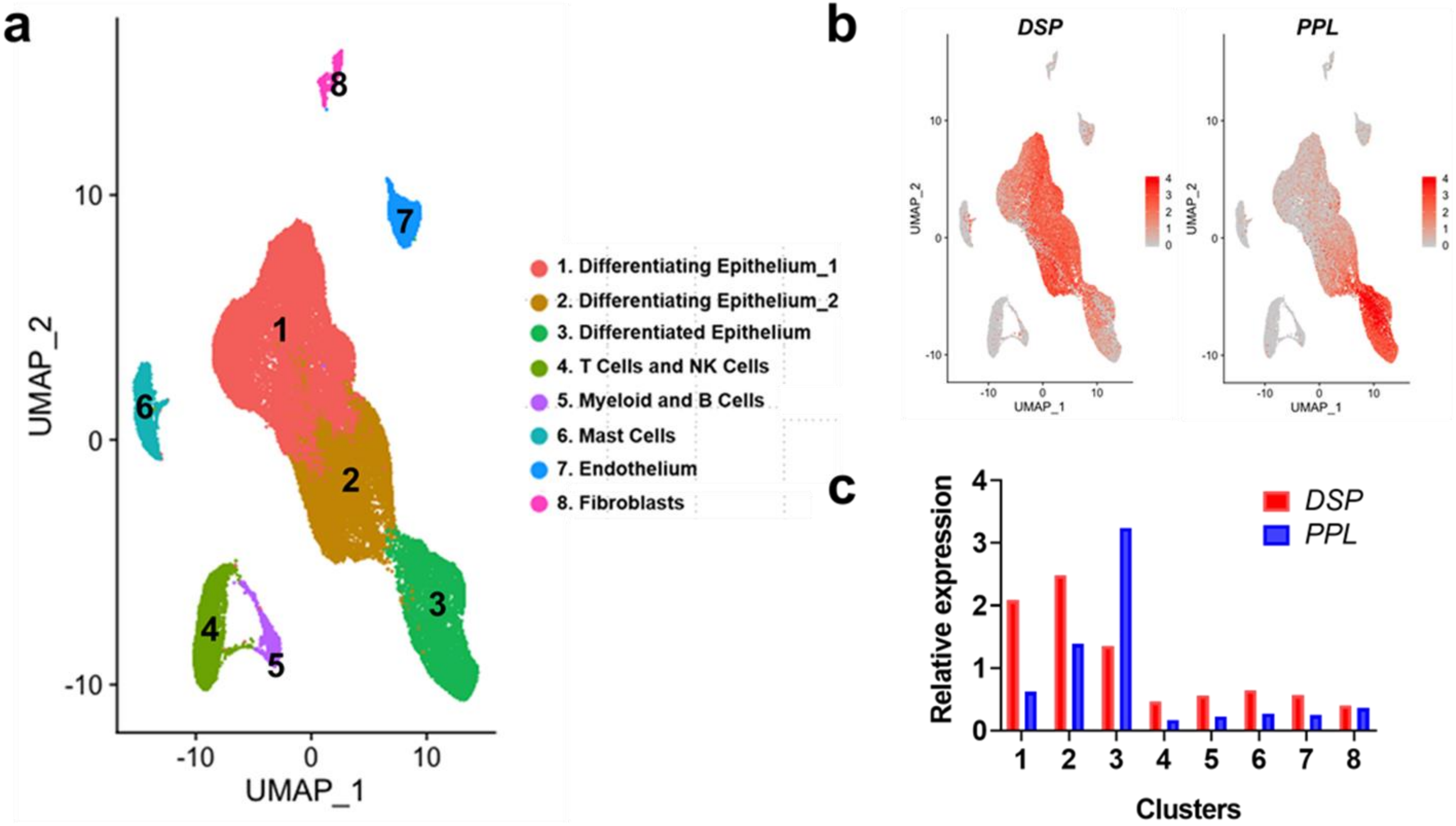
a
b
c
UMAP_1
UMAP_2
1
2
3
4
5
6
7
8
1. Differentiating Epithelium_1
2. Differentiating Epithelium_2
3. Differentiated Epithelium
4. T Cells and NK Cells
5. Myeloid and B Cells
6. Mast Cells
7. Endothelium
8. Fibroblasts
DSP
PPL
Relative expression
Clusters

Supplementary Figure 7

DSP and *PPL* expression in epithelial cell populations.

a, Uniform manifold approximation and projection (UMAP) plot displaying single cells, colored by shared nearest neighbor clusters and cell types from a single-cell RNA-sequencing analysis of esophageal biopsies. **b,** Feature plots demonstrating the expression of *DSP* and *PPL*. Each dot represents a single cell. **c,** Relative expressions of *DSP* and *PPL* based on single-cell RNA–sequencing data; cluster numbers match those shown in panel **a**.

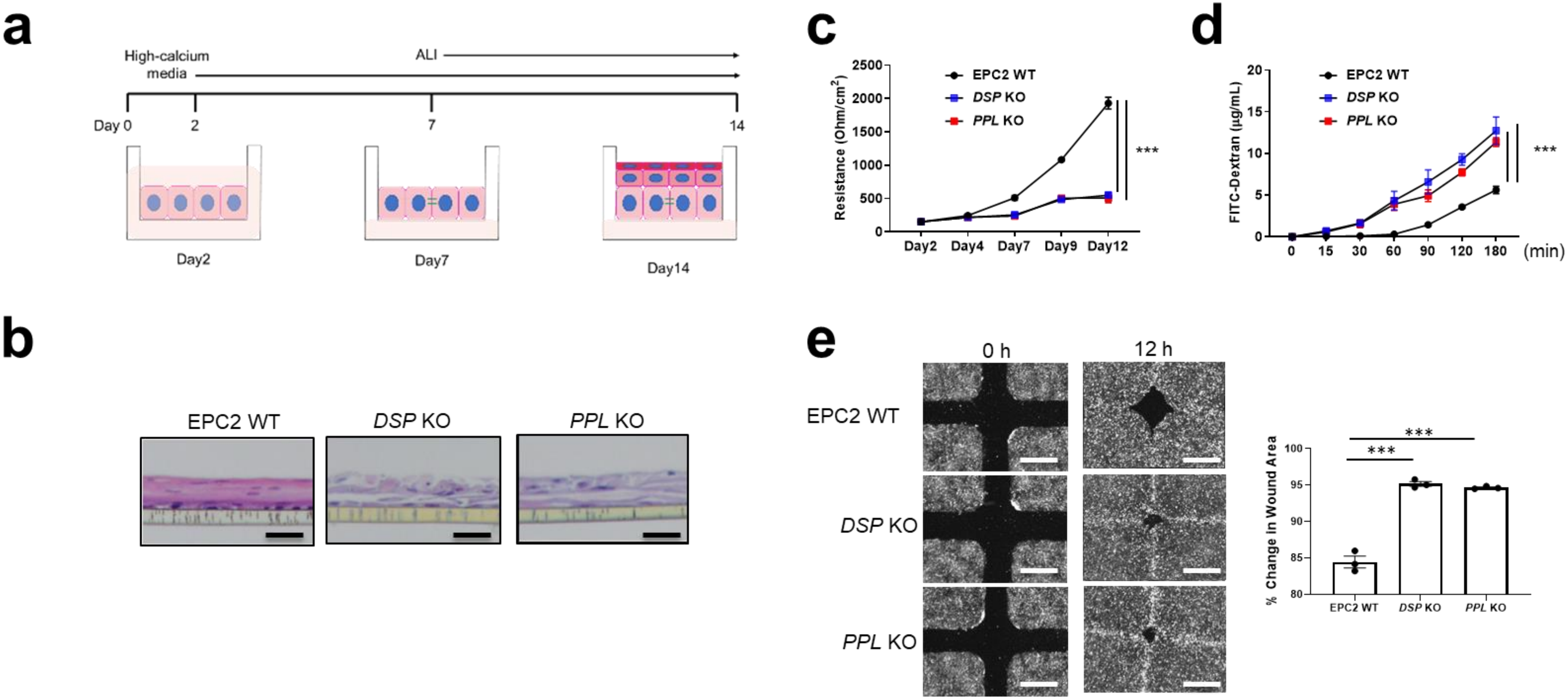
a
High-calcium media
ALI
Day 0
2
7
14
Day2
Day7
Day14
b
EPC2 WT
DSP KO
PPL KO
c
EPC2 WT
DSP KO
PPL KO
Resistance (Ohm/cm²)
2500
2000
1500
1000
500
0
Day2
Day4
Day7
Day9
Day12
***
d
EPC2 WT
DSP KO
PPL KO
FITC-Dextran (μg/mL)
20
15
10
5
0
0
15
30
60
90
120
180
(min)
***
e
0 h
12 h
EPC2 WT
DSP KO
PPL KO
% Change in Wound Area
100
95
90
85
80
EPC2 WT
DSP KO
PPL KO
***
***

Supplementary Figure 8

Effect of loss of DSP and PPL on epithelial differentiation, barrier function and cell motility.

a, Schematic outline of the differentiation protocol for EPC2 cells grown at the air-liquid interface (ALI). **b,** Hematoxylin and eosin (H&E)–stained sections of either WT, *DSP* KO, or *PPL* KO EPC2 cells after ALI differentiation (day 14). Scale bar: 50 μM. **c,** the transepithelial electrical resistance (TEER) and **d,** FITC-dextran flux measurements for EPC2 cells grown at the ALI. Statistics: **c**, EPC2 WT vs. DSP KO, P < 0.0001; EPC2 WT vs. PPL KO, P < 0.0001; **d**, EPC2 WT vs. DSP KO, P < 0.0001; EPC2 WT vs. PPL KO, P < 0.0001. **e,** Wound healing assays simultaneously performed for WT, *DSP* KO and *PPL* KO EPC2 cells. Representative image of the wound healing assay at the assay start (0 h) and completion (12 h) time points and quantification of the wound closure after 12 h was shown. Scale bar: 500 μM. Statistics: EPC2 WT vs. DSP KO, P < 0.0001; EPC2 WT vs. PPL KO, P < 0.0001; DSP KO vs. PPL KO, P = 0.759. For panels **b-e**, data are representative of three experiments performed in duplicate. For panels **c-e**, data are presented as mean ± SEM, and two-tailed P values were determined by the two-way ANOVA test followed by a Holm-Sidak's multiple comparisons test (**c-d)** or one-way ANOVA test followed by a Dunnett’s multiple-comparison test (**e**). *P < 0.05, **P < 0.01 and ***P < 0.001. WT, wild-type; KO, knockout; MW, molecular weight marker; SEM, standard error of the mean.

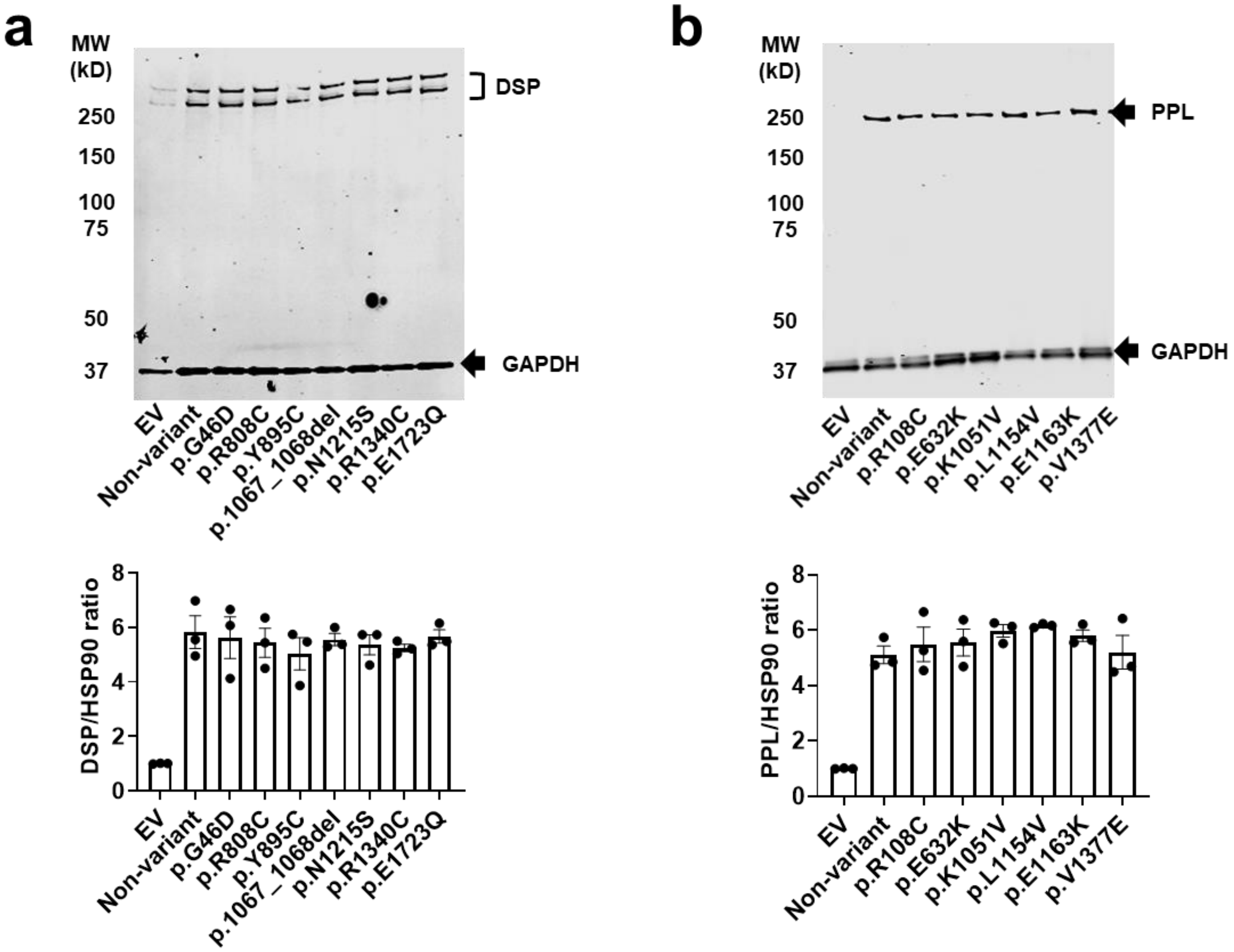
a
MW (kD)
250
150
100
75
50
37
DSP
GAPDH
EV
Non-variant
p.G46D
p.R808C
p.Y895C
p.1067_1068del
p.N1215S
p.R1340C
p.E1723Q
DSP/HSP90 ratio
0
2
4
6
8
b
MW (kD)
250
150
100
75
50
37
PPL
GAPDH
EV
Non-variant
p.R108C
p.E632K
p.K1051V
p.L1154V
p.E1163K
p.V1377E
PPL/HSP90 ratio
0
2
4
6
8

Supplementary Figure 9

Expression of all mutant DSP and PPL constructs in transduced cells.

a-b, Representative western blots of ectopic expression of DSP (**a**) and PPL (**b**) proteins for each construct with anti-DSP or anti-PPL antibody (top) and anti-HSP90 antibody (bottom) as a loading control. Data are representative of three independent experiments performed in duplicate and are presented as mean ± SEM. EV, empty vector; MW, molecular weight marker; SEM, standard error of the mean.

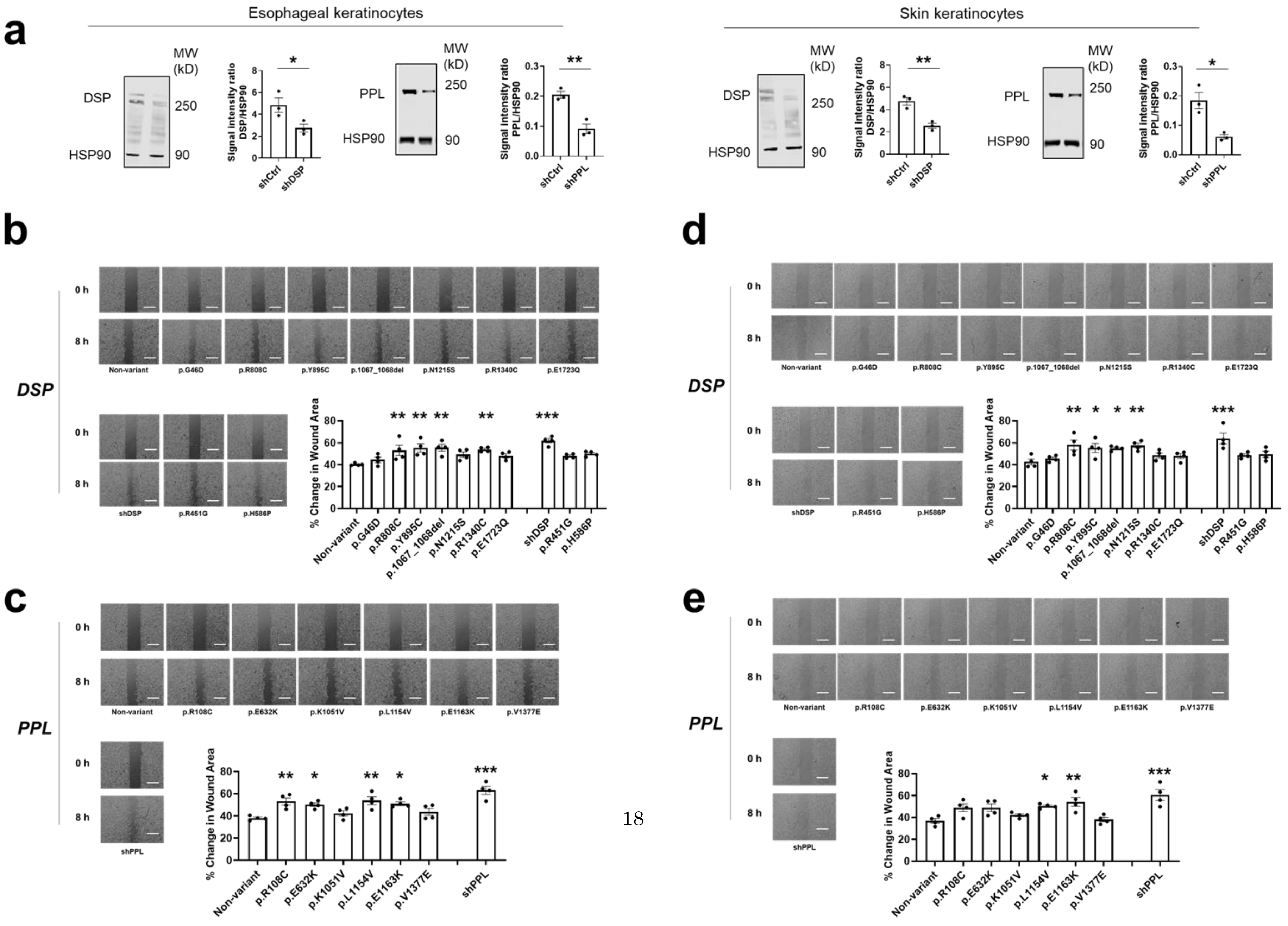
a
Esophageal keratinocytes
Skin keratinocytes
MW (kD)
DSP
HSP90
PPL
250
90
Signal intensity ratio DSP/HSP90
Signal intensity ratio PPL/HSP90
shCtrl
shDSP
shPPL
b
c
d
e
DSP
PPL
0 h
8 h
Non-variant
p.G46D
p.R808C
p.Y895C
p.1067_1068del
p.N1215S
p.R1340C
p.E1723Q
shDSP
p.R451G
p.H586P
p.R108C
p.E632K
p.K1051V
p.L1154V
p.E1163K
p.V1377E
shPPL
% Change in Wound Area

Supplementary Figure 10

Mutational effect of *DSP* and *PPL* on wound healing assay.

a, Representative western blot of DSP or PPL after downregulation by shRNA in esophageal (left) or skin keratinocytes (right). HSP90 serves as a loading control. Statistics: esophageal keratinocytes (shDSP vs. shCtrl, P = 0.0499; shPPL vs. shCtrl, P = 0.0046); skin keratinocytes (shDSP vs. shCtrl, P = 0.0054; shPPL vs. shCtrl, P = 0.0126). **b–e,** Representative images of the wound area at assay start (0 h) and completion (8 h) time points. Cells [esophageal (**b** and **c**) or skin keratinocytes (**d** and **e**)] were transduced with *DSP* (**b** and **d**) or *PPL* (**c** and **e**) constructs encoding non-variant and mutants. shDSP, p.R451G, p.H586P and shPPL are controls. Scale bar: 500 µM. Statistics (versus non-variants): **b** (p.G46D, P = 0.8239; p.R808C, P = 0.0077; p.Y895C, P = 0.0015; p.1067_1068del, P = 0.0012; p.N1215S, P = 0.107; p.R1340C, P = 0.0042; p.E1723Q, P = 0.1907; shDSP, P < 0.0001; p.R451G, P = 0.2205; p.H586P, P = 0.0677); **c** (p.R108C, P = 0.0032; p.E632K, P = 0.0212; p.K1051V, P = 0.7612; p.L1154V, P = 0.002; p.E1163K, P = 0.0124; p.V1377E, P = 0.5217; shPPL, P < 0.0001); **d** (p.G46D, P = 0.9867; p.R808C, P = 0.0066; p.Y895C, P = 0.0335; p.1067_1068del, P = 0.0409; p.N1215S, P = 0.0095; p.R1340C, P = 0.6667; p.E1723Q, P = 0.7629; shDSP, P < 0.0001; p.R451G, P = 0.6324; p.H586P, P = 0.4854); **e** (p.R108C, P = 0.0568; p.E632K, P = 0.0548; p.K1051V, P = 0.7427; p.L1154V, P = 0.0274; p.E1163K, P = 0.0035; p.V1377E, P = 0.9996; shPPL, P = 0.0001). For panels **a-e**, data are representative of three experiments performed in duplicate and are presented as mean ± SEM. For panels **a-e**, two-tailed P values were determined by the unpaired t test (**a**) or one-way ANOVA test followed by a Dunnett's multiple-comparison test (**b-e**). *P < 0.05, **P < 0.01 and ***P < 0.001. DSP, desmoplakin; PPL, periplakin; SEM, standard error of the mean.

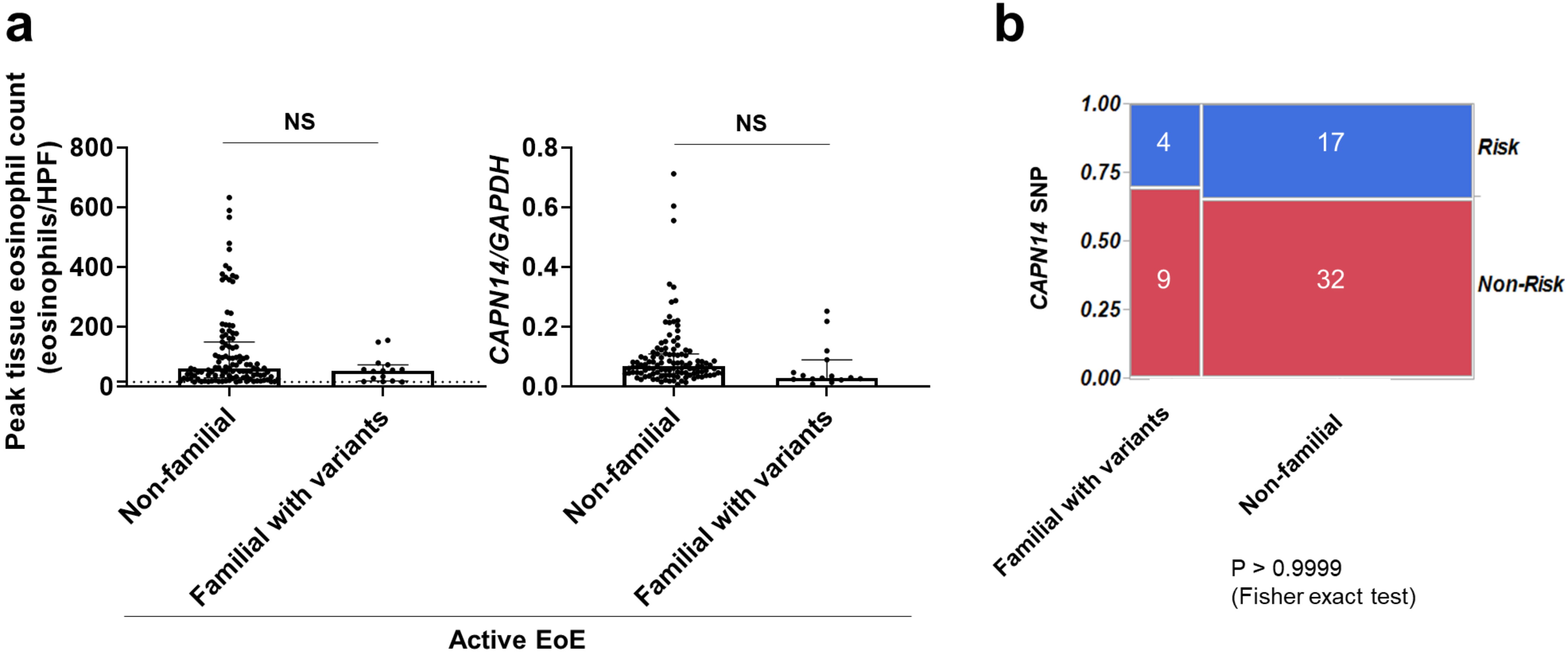
a
Peak tissue eosinophil count (eosinophils/HPF)
NS
800
600
400
200
0
Non-familial
Familial with variants
CAPN14/GAPDH
NS
0.8
0.6
0.4
0.2
0.0
Non-familial
Familial with variants
Active EoE
b
CAPN14 SNP
1.00
0.75
0.50
0.25
0.00
4
17
9
32
Risk
Non-Risk
Familial with variants
Non-familial
P > 0.9999
(Fisher exact test)

Supplementary Figure 11

***CAPN14* SNP and gene expression in patients with variants in *DSP* and/or *PPL*.**

a, Peak esophageal eosinophil counts (left) and *CAPN14* mRNA expression (right) are plotted by groups for non-familial and familial EoE with *DSP* and/or *PPL* variants (non-familial EoE, n = 115; familial EoE with variants, n = 15). All samples were from the biopsies during the active disease state. The dashed line indicates the diagnostic threshold of EoE (15 eosinophil/HPF). Data are presented as mean ± SEM, with markers representing individual subjects. Statistics: peak esophageal eosinophil count, P = 0.0927; *CAPN14*, P = 0.2326. **b,** *CAPN14* SNP in patients with variants in *DSP* and/or *PPL*. The mosaic plot including the number of patients represents the two-way frequency. The numbers in each box indicate the number of subjects. Statistics: P > 0.9999. For panels **a-b**, two-tailed P values were determined by the unpaired t test (**a**) or Fisher's exact test (**b**). *CAPN14*, calpain-14; *DSP*, desmoplakin; *GAPDH*, glyceraldehyde-3-phosphate dehydrogenase; HPF, high-power microscopic field; NS, not significant; *PPL*, periplakin; SNP, single nucleotide polymorphism; SEM, standard error of the mean.

Supplementary Table 1. Details of variants meeting filtering criteria

Gene Symbol	Chr	Position	Ref	Alt	Protein	cDNA	Annotation	MAF*	Sanger sequencing					Esophageal tissue expression (TPM)#
									F430.1	F430.2	F430.4	F430.11	F430.19	
LRP2	2	170131729	C	T	p.G598S	c.G1792A	Missense	1.06E-04	m/+	m/+	m/+	m/+	m/+	0.006
DSP	6	7542285	G	A	p.G46D	c.G137A	Missense	8.43E-05	m/+	m/+	m/+	m/+	m/+	647.4
HIST1H1C	6	26056587	C	T	p.A24T	c.G70A	Missense	3.22E-04	m/+	+/+	+/+	m/+	m/+	67.2
ZKSCAN4	6	28219674	T	C	p.K29E	c.A85G	Missense	9.93E-05	m/+	m/+	m/+	m/+	m/+	4.0
ZNF174	16	3458792	A	G	p.Q366R	c.A1097G	Missense	3.00E-05	m/+	m/+	+/+	m/+	+/+	5.5
PPL	16	4934526	A	T	p.V1377E	c.T4130A	Missense	1.38E-04	m/+	m/+	m/+	m/+	m/+	1087.0

*Minor allele frequencies (MAF) from the exomes in the ExAC Database (https://gnomad.broadinstitute.org/)

#Normal tissue expression from the GTEx Database (https://www.gtexportal.org/home/).

Abbreviations: Chr, chromosome; Ref, reference nucleotide; Alt, alternate nucleotide; MAF, minor allele frequency; m, presence of mutant allele; +, reference allele; TPM, transcripts per kilobase million.

Supplementary Table 2. Minor allele frequencies (MAF) from ExAC for all *DSP* and *PPL* variants included in this study*

Family	Gene Symbol	Chr	Position	Ref	Alt	Protein	cDNA	Annotation	ALL	African	East Asian	European (Non-Finnish)	Finnish	Latino	Other	South Asian
430	*DSP*	6	7542285	G	A	p.G46D	c.G137A	missense	3.80E-05	0	0	8.40E-05	0	0	0	0
305	*DSP*	6	7575014	C	T	p.R808C	c.C2422T	missense	3.70E-04	0	1.20E-04	6.00E-04	1.50E-04	0	0	1.80E-04
964	*DSP*	6	7576580	A	G	p.Y895C	c.A2684G	missense	2.00E-04	0	0	1.00E-04	0	6.00E-04	1.10E-03	5.50E-04
2285	*DSP*	6	7579624	AG	-	p.1067_1068del	c.3201_3202del	frameshift deletion	-	-	-	-	-	-	-	-
999	*DSP*	6	7580067	A	G	p.N1215S	c.A3644G	missense	8.30E-06	0	0	1.50E-05	0	0	0	0
471	*DSP*	6	7580441	C	T	p.R1340C	c.C4018T	missense	8.30E-05	0	2.30E-04	1.10E-04	0	0	1.10E-03	0
1594	*DSP*	6	7581590	G	C	p.E1723Q	c.G5167C	missense	2.20E-04	9.70E-05	0	3.60E-04	0	8.70E-05	0	0
179, 186	*PPL*	16	4952523	G	A	p.R108C	c.C322T	missense	2.50E-05	0	0	0	0	0	0	1.90E-04
387	*PPL*	16	4941886	C	T	p.E632K	c.G1894A	missense	1.20E-04	9.60E-05	0	9.00E-05	0	0	1.10E-03	3.70E-04
1176	*PPL*	16	4935504_ 4935505	TT	AC	p.K1051V	c.3151_3152del insGT	deletion-insertion	-	-	-	-	-	-	-	-
1029	*PPL*	16	4935196	G	C	p.L1154V	c.C3460G	missense	-	-	-	-	-	-	-	-
44	*PPL*	16	4935169	C	T	p.E1163K	c.G3487A	missense	8.20E-06	0	0	1.50E-05	0	0	0	0
430	*PPL*	16	4934526	A	T	p.V1377E	c.T4130A	missense	7.60E-05	0	0	1.40E-04	0	0	0	0

*Minor allele frequencies (MAF) for all variants observed in our index cases, from the exomes in the ExAC Database (https://gnomad.broadinstitute.org/).

Abbreviations: Chr, chromosome; Ref, reference nucleotide; Alt, alternate nucleotide; ALL, all population; ExAC, Exome Aggregation Consortium.

Supplementary Table 3. Algorithms predicting molecular pathogenicity for all *DSP* and *PPL* variants included in this study*

Gene Symbol	Chr	Position	Protein	cDNA	SIFT	PolyPhen2 HDIV	PolyPhen2 HVAR	LRT	Mutation Taster	Mutation Assessor	FATHMM	CADD	Radial SVM	LR
DSP	6	7542285	p.G46D	c.G137A	0.83 (T)	0.01 (B)	0.007 (B)	0.566 (N)	0 (N)	0.481 (N)	0.426 (T)	12.7 (N)	0.241 (T)	0.069 (T)
DSP	6	7575014	p.R808C	c.C2422T	0.78 (T)	0.484 (P)	0.047 (B)	1 (D)	1 (D)	0.548 (N)	0.468 (D)	26.4 (D)	0.367 (T)	0.264 (T)
DSP	6	7576580	p.Y895C	c.A2684G	0.95 (D)	0.998 (D)	0.747 (P)	1 (D)	0.993 (D)	0.607 (L)	0.346 (T)	25 (D)	0.243 (T)	0.088 (T)
DSP	6	7579624	p.1067_1068del	c.3201_3202del	NA	NA	NA	NA	NA	NA	NA	35 (D)	NA	NA
DSP	6	7580067	p.N1215S	c.A3644G	0.32 (T)	0.015 (B)	0.007 (B)	1 (D)	1 (D)	0.541 (N)	0.502 (D)	18.2 (D)	0.321 (T)	0.287 (T)
DSP	6	7580441	p.R1340C	c.C4018T	0.95 (D)	0.978 (D)	0.451 (P)	1 (D)	1 (D)	0.611 (L)	0.527 (D)	27.7 (D)	0.585 (D)	0.704 (D)
DSP	6	7581590	p.E1723Q	c.G5167C	0.86 (T)	1 (D)	0.996 (D)	1 (D)	1 (D)	0.625 (L)	0.438 (T)	25.5 (D)	0.474 (T)	0.53 (D)
PPL	16	4952523	p.R108C	c.C322T	1 (D)	1 (D)	0.996 (D)	1 (D)	1 (D)	0.659 (M)	0.349 (T)	29.4 (D)	0.336 (T)	0.219 (T)
PPL	16	4941886	p.E632K	c.G1894A	1 (D)	0.944 (P)	0.296 (B)	1 (D)	1 (D)	0.67 (M)	0.366 (T)	23.1 (D)	0.328 (T)	0.162 (T)
PPL	16	4935504_4935505	p.K1051V	c.3151_3152delinsGT	0.99 (D)	0.846 (P)	0.527 (P)	NA	NA	NA	NA	16.3 (D)	NA	NA
PPL	16	4935169	p.L1154V	c.C3460G	0.92 (T)	0.997 (D)	0.888 (P)	1 (D)	1 (D)	0.696 (M)	0.409 (T)	24.1 (D)	0.468 (T)	0.439 (T)
PPL	16	4935196	p.E1163K	c.G3487A	0 (T)	0.757 (P)	0.293 (B)	1 (D)	0.999 (D)	0.581 (L)	0.367 (T)	22.9 (D)	0.246 (T)	0.089 (T)
PPL	16	4934526	p.V1377E	c.T4130A	0 (T)	0.01 (B)	0.018 (B)	0.025 (N)	0 (N)	0.411 (N)	0.348 (T)	0.32 (N)	0.254 (T)	0.026 (T)

*Categorical predictions for each algorithm are summarized in Table S5. SIFT: "T"=Tolerated, "D"=Damaging. PolyPhen2 HDIV & HVAR: "B"=Benign, "P"=Possibly Damaging, "D"=Probably Damaging. LRT: "N"=Neutral, "D"=Damaging. Mutation Taster: "N"=Polymorphisms, "D"=Disease-causing. Mutation Assessor: "M"=Functional, "L" & "N"=Non-functional. FATHMM: "T"=Tolerated, "D"=Deleterious. CADD: "N"=Neutral, "D"= Deleterious. Radial SVM: "T"=Tolerated, "D"=Damaging. LR: "T"=Tolerated, "D"=Damaging. Abbreviations: Chr, chromosome; NA, not applicable.

Supplementary Table 4. Algorithms predicting amino acid evolutionary conservation and nucleotide conservation for all *DSP* and *PPL* variants included in this study

Gene Symbol	Chr	Position	Protein	cDNA	Multiz-Alignment Mammals*	GERP++[#]	PhyloP[#]	SiPhy[#]
DSP	6	7542285	p.G46D	c.G137A	6 (60)	0.7 (NC)	-0.1 (NC)	3.8 (NC)
DSP	6	7575014	p.R808C	c.C2422T	0 (62)	5.8 (C)	2.8 (C)	14.9 (C)
DSP	6	7576580	p.Y895C	c.A2684G	0 (61)	6.0 (C)	2.3 (C)	12.4 (C)
DSP	6	7579624	p.1067_1068del	c.3201_3202del	NA	NA	NA	NA
DSP	6	7580067	p.N1215S	c.A3644G	1 (62)	4.2 (NC)	0.9 (NC)	7.0 (NC)
DSP	6	7580441	p.R1340C	c.C4018T	0 (62)	5.5 (C)	2.6 (C)	14.2 (C)
DSP	6	7581590	p.E1723Q	c.G5167C	0 (62)	5.9 (C)	2.8 (C)	20.2 (C)
PPL	16	4952523	p.R108C	c.C322T	2 (61)	5.0 (C)	2.3 (C)	16.4 (C)
PPL	16	4941886	p.E632K	c.G1894A	0 (60)	5.4 (C)	2.5 (C)	19.1 (C)
PPL	16	4935504_4935505	p.K1051V	c.3151_3152del insGT	NA	NA	NA	NA NA
PPL	16	4935196	p.L1154V	c.C3460G	0 (62)	5.2 (C)	2.4 (C)	12.1 (NC)
PPL	16	4935169	p.E1163K	c.G3487A	0 (62)	4.9 (C)	2.3 (C)	14.6 (C)
PPL	16	4934526	p.V1377E	c.T4130A	0 (62)	-7.2 (NC)	-1.5 (NC)	6.9 (NC)

*The Multiz-Alignment counts the number of mammalian species included in the UCSC Genome Browser whose reference allele matches the observed amino acid variant. [#]Algorithms (PhyloP, SiPhy and GERP++) include information concerning known evolutionary conservation at the level of DNA base pairs. Categorical predictions for each algorithm are summarized in Table S5. "C"=Conserved, "NC"=Not Conserved. Abbreviations: NA, not applicable.

Supplementary Table 5. Summary of deleteriousness prediction methods analyzed in this study

Name	Category	Deleterious threshold*	Information used
SIFT	Function prediction	>0.95	Protein sequence conservation among homologs
PolyPhen-2	Function prediction	>0.5	Eight protein sequence features, three protein structure features
LRT	Function prediction	>0.999	DNA sequence evolutionary model
Mutation Taster	Function prediction	>0.5	DNA sequence conservation, splice site prediction, mRNA stability prediction and protein feature annotations project and information from UCSC genome browser tracks
Mutation Assessor	Function prediction	>0.65	Sequence homology of protein families and sub-families within and between species
FATHMM	Function prediction	≥0.45	Sequence homology
CADD	Ensemble score	>15	63 distinct variant annotation retrieved from Ensembl Variant Effect Predictor (VEP), data from the ENCODE project and information from UCSC genome browser tracks
RadialSVM	Ensemble score	>0.5	Ensemble-based approach integrating multiple scoring systems by radial support vector machine
LR score	Ensemble score	>0.5	Ensemble-based approach integrating multiple scoring systems by logistic regression
GERP++ RS	Conservation score	>4.4	DNA sequence conservation
PhyloP	Conservation score	>1.6	DNA sequence conservation
SiPhy	Conservation score	>12.17	Inferred nucleotide substitution pattern per site

*Score indicates raw score for the corresponding function prediction/conservation score output.

Supplementary Table 6. Case-control association studies for all *DSP* and *PPL* variants included in this study

Gene Symbol	Chr	Position	Protein	cDNA	EoE		Controls		Odds ratio	95% CI	P value
					Minor allele	Major allele	Minor allele	Major allele			
DSP	6	7542285	p.G46D	c.G137A	1	123	1	11870	96.50	6.00–1551.67	0.02
DSP	6	7575014	p.R808C	c.C2422T	1	123	40	66724	13.56	1.85–99.43	0.07
DSP	6	7576580	p.Y895C	c.A2684G	1	123	7	66726	77.50	9.46–634.61	0.02
DSP	6	7579624	p.1067_1068del	c.3201_3202del	1	123	-	-	-	-	-
DSP	6	7580067	p.N1215S	c.A3644G	1	123	1	66240	538.54	33.50–8658.59	0.004
DSP	6	7580441	p.R1340C	c.C4018T	1	123	7	66322	77.03	9.41–630.76	0.02
DSP	6	7581590	p.E1723Q	c.G5167C	1	123	24	66428	22.50	3.02–167.64	0.05
PPL	16	4952523	p.R108C	c.C322T	2	122	0	66320	∞	-	3.5E-06
PPL	16	4941886	p.E632K	c.G1894A	1	123	6	66346	89.90	10.74–752.26	0.01
PPL	16	4935504_4935505	p.K1051V	c.3151_3152delinsGT	1	123	-	-	-	-	-
PPL	16	4935196	p.L1154V	c.C3460G	1	123	-	-	-	-	-
PPL	16	4935169	p.E1163K	c.G3487A	1	123	1	66716	542.41	33.74–8720.81	0.004
PPL	16	4934526	p.V1377E	c.T4130A	1	123	9	65368	59.04	7.43–469.62	0.02

Observed allele counts for all variants screened in the index cases and the combined exomes of population-matched allele counts in the ExAC Database (https://gnomad.broadinstitute.org/). The odds ratio (OR) with 95% confidence interval (95% CI) using the chi-square test and statistical significance (two-tailed P-values) using the Fisher's exact test were shown. Abbreviations: Chr, chromosome; EoE, eosinophilic esophagitis.

Supplementary Table 7. Details of other 7 rare desmosomal gene variants in subjects with isolated *DSP* and *PPL* rare variants

Gene symbol	Chr	Position	Ref	Alt	cDNA	Protein	Minor allele frequencies (ExAC)
CDSN	6	31084974	G	A	c.C418T	p.H140Y	-
DSG1	18	28923439	G	A	c.G1714A	p.D572N	5.8E-04
EVPL	17	74007868	C	G	c.G2553C	p.E851D	1.0E-04
JUP	17	39925350	A	G	c.T578C	p.M193T	4.3E-05
PERP	6	138417514	A	G	c.T332C	p.I111T	5.8E-05
PERP	6	138428303	C	T	c.G175A	p.G59R	2.7E-03
PKP3	11	400007	C	G	c.C1314G	p.D438E	-

Gene symbol	CADD	SIFT	Polyphen 2_HDIV	Polyphen 2_HVAR	LRT	Mutation Taster	Mutation Assessor	FATHMM	Radial SVM	LR
CDSN	12.2 (N)	0.94 (T)	0.775 (P)	0.101 (B)	0.66 (N)	0 (N)	0.541 (N)	0.269 (T)	0.267 (T)	0.005 (T)
DSG1	22.3 (D)	0.98 (D)	0.496 (P)	0.245 (B)	0.987 (N)	1 (D)	0.681 (M)	0.371 (T)	0.305 (T)	0.168 (T)
EVPL	5.7 (N)	0.68 (T)	0.002 (B)	0.004 (B)	0.994 (N)	0 (N)	0.583 (L)	0.394 (T)	0.24 (T)	0.107 (T)
JUP	23.8 (D)	0.74 (T)	0.993 (D)	0.987 (D)	1 (D)	0.999 (D)	0.603 (L)	0.372 (T)	0.301 (T)	0.227 (T)
PERP	26.9 (D)	0.99 (D)	1 (D)	0.998 (D)	1 (D)	1 (D)	0.612 (L)	0.477 (D)	0.578 (D)	0.668 (D)
PERP	23.4 (D)	0.51 (T)	1 (D)	0.999 (D)	0.825 (N)	1 (D)	0.511 (N)	0.489 (D)	0.467 (T)	0.536 (D)
PKP3	15.3 (D)	0 (T)	0.003 (B)	0.002 (B)	0.998 (N)	0.606 (D)	0.385 (N)	0.428 (T)	0.266 (T)	0.089 (T)

*Categorical predictions for each algorithm are summarized in Table S5. SIFT: "T"=Tolerated, "D"=Damaging. PolyPhen2 HDIV & HVAR: "B"=Benign, "P"=Possibly Damaging, "D"=Probably Damaging. LRT: "N"=Neutral, "D"=Damaging. Mutation Taster: "N" =Polymorphisms, "D"=Disease-causing. Mutation Assessor: "M"=Functional, "L" & "N"=Non-functional. FATHMM: "T"=Tolerated, "D"=Deleterious. CADD: categorical algorithm predicting molecular as described in Table S5; "N"=Neutral, "D"= Deleterious. Radial SVM: "T"=Tolerated, "D"=Damaging. LR: "T"=Tolerated, "D"=Damaging.

Supplementary Table 8. Patient demographics examined by molecular expression analysis and histologic analysis*

	A. Histologic analysis					B. Molecular expression analysis				Correlation
		Non-familial EoE		Familial EoE with variants			Non-familial EoE		Familial EoE with variants (Active)	Subjects having A & B
	Control	Inactive EoE	Active EoE	Inactive EoE	Active EoE	Control	Inactive EoE	Active EoE		
	N = 58	N = 254	N = 271	N = 14	N = 7	N = 48	N = 51	N = 132	N = 15	N = 68
Age at collection	11.7 (7.3 - 15.8)	10.9 (6.2 - 14.9)	10.4 (6.2 - 14.3)	13.8 (9.4 - 17.9)	15.4 (12.4 - 16.7)	10.0 (4.4 - 15.5)	8.1 (5.2 - 12.8)	9.7 (5.3 - 15.3)	10.1 (6.9 - 13.7)	10.3 (5.3 - 18.0)
Male	21 (36.2%)	197 (77.6%)	218 (80.4%)	8 (57.1%)	4 (57.1%)	29 (60.4%)	46 (90.2%)	102 (77.3%)	10 (66.7%)	50 (73.5%)
White/Caucasian	53 (91.4%)	240 (94.5%)	249 (91.9%)	7 (50.0%)	7 (100.0%)	47 (97.9%)	49 (96.1%)	125 (94.7%)	14 (93.3%)	63 (92.6%)
**Tissue eosinophil counts**										
Peak (eos/hpf)	0 (0 - 0)	1 (0 - 4)	42 (24 - 87)	0 (0 - 2.8)	27 (18 - 177)	0 (0 - 0)	3 (0 - 10)	59 (27.5 - 148.3)	52 (18 - 72)	51 (19 - 153.3)
Range (min. – max.)	(0 - 2)	(0 - 14)	(15 - 576)	(0 - 6)	(17 - 332)	(0 - 2)	(0 - 14)	(15 - 632)	(15 - 154)	(0 - 632)
**Atopic status**										
Atopy	30 (51.7%)	192 (75.6%)	213 (78.6%)	13 (92.9%)	6 (85.7%)	26 (54.2%)	45 (88.2%)	110 (83.3%)	13 (86.7%)	56 (82.4%)
Asthma	16 (27.6%)	97 (38.2%)	117 (43.2%)	7 (50.0%)	4 (57.1%)	14 (29.2%)	23 (45.1%)	56 (42.4%)	7 (46.7%)	31 (45.6%)
Allergic rhinitis	14 (24.1%)	132 (52.0%)	149 (55.0%)	9 (64.3%)	3 (42.9%)	17 (35.4%)	35 (68.6%)	86 (65.2%)	8 (53.3%)	39 (57.4%)
Eczema	15 (25.9%)	132 (52.0%)	140 (51.7%)	5 (35.7%)	4 (57.1%)	11 (22.9%)	28 (54.9%)	67 (50.8%)	5 (33.3%)	33 (48.5%)
Anaphylaxis by food	2 (3.4%)	68 (26.8%)	58 (21.4%)	6 (42.9%)	3 (42.9%)	4 (8.3%)	21 (41.2%)	30 (22.7%)	4 (26.7%)	9 (13.2%)
**Treatment at biopsy**										
Ongoing diet therapy	10 (17.2%)	180 (70.9%)	164 (60.5%)	11 (78.6%)	5 (71.4%)	13 (27.1%)	42 (82.4%)	89 (67.4%)	8 (53.3%)	40 (58.8%)
PPI	32 (55.2%)	173 (68.1%)	161 (59.4%)	11 (78.6%)	5 (71.4%)	16 (33.3%)	34 (66.7%)	89 (67.4%)	11 (73.3%)	47 (69.1%)
Topical steroids	1 (1.7%)	127 (50.0%)	95 (35.1%)	8 (57.1%)	3 (42.9%)	1 (2.1%)	26 (51.0%)	40 (30.3%)	3 (20.0%)	14 (20.6%)
Systemic steroids	0 (0%)	2 (0.8%)	3 (1.1%)	0 (0%)	0 (0%)	0 (0%)	1 (2.0%)	2 (2.3%)	0 (0%)	1 (1.5%)

*Data are n (%) or median (interquartile range [IQR]) unless otherwise stated. EoE, eosinophilic esophagitis; eos/hpf, eosinophils per high-power field; PPI, proton pump inhibitor.

Supplementary Table 9. Comparison of histologic features of control, non-familial EoE and familial EoE

Comparison	EoE histology scoring system (HSS)*										
	Total Score	Eosinophil feature	Structural feature	EI	EA	ESL	SEA	BZH	DIS	DEC	LPF
Global	< 0.0001	< 0.0001	< 0.0001	< 0.0001	< 0.0001	< 0.0001	< 0.0001	< 0.0001	< 0.0001	0.8003	< 0.0001
Control vs Non-familial EoE (inactive)	0.0497	0.008	0.046	0.0398	1	1	1	0.0615	0.5779	1	1
Control vs Non-familial EoE (active)	< 0.0001	< 0.0001	< 0.0001	< 0.0001	< 0.0001	< 0.0001	0.0011	< 0.0001	< 0.0001	1	0.0073
Control vs Familial EoE (inactive)	0.0741	1	0.005	1	1	1	1	0.397	0.0021	1	0.0197
Control vs Familial EoE (active)	< 0.0001	< 0.0001	< 0.0001	< 0.0001	0.0064	0.0044	0.0002	< 0.0001	0.0063	1	0.3932
Non-familial EoE (inactive) vs Non-familial EoE (active)	< 0.0001	< 0.0001	< 0.0001	0.0001	< 0.0001	< 0.0001	0.0008	< 0.0001	< 0.0001	1	< 0.0001
Non-familial EoE (inactive) vs Familial EoE (inactive)	1	1	0.2287	1	1	1	1	1	0.0258	1	0.0124
Non-familial EoE (inactive) vs Familial EoE (active)	< 0.0001	< 0.0001	0.0006	< 0.0001	0.0036	0.0038	0.0008	0.0007	0.0437	1	0.484
Non-familial EoE (active) vs Familial EoE (inactive)	0.0006	< 0.0001	0.5017	0.0008	0.0348	0.1148	1	0.0007	1	1	1
Non-familial EoE (active) vs Familial EoE (active)	1	1	1	1	1	0.6213	0.0231	1	0.9009	1	1
Familial EoE (inactive) vs Familial EoE (active)	0.0264	0.0004	0.4948	< 0.0001	0.0318	0.0237	0.0402	0.0449	1	1	1

*P values by the Kruskal-Wallis test followed by a Dunn multiple-comparison test.

Abbreviations: EoE, eosinophilic esophagitis; EI, eosinophilic inflammation; EA, eosinophilic abscess; ESL, eosinophilic surface layering; SEA, surface epithelial alteration; BZH, basal zone hyperplasia; DIS, dilated intercellular spaces; DEC, dyskeratotic epithelial cells; LPF, lamina propria fibrosis.

Supplementary Table 10. List of primers used for Sanger sequencing

Name	Forward	Reverse
DSP_c.G137A	GTAGCGAGCAGCGACCTC	GAGTCTCCCAGTCGTCCAGA
DSP_c.C2422T	CAGTAAGGGCACTGCTCCAG	GTGGCCACTGAAACCCAATA
DSP_c.A2684G	TCAAGTGAATTTCTGGGTGATTC	ACAGCTATGGAACAAAAAGTTGG
DSP_c.3201_3202del	TGATTTCATTCCACAGCTGAA	ATCTCATAAGTCAGTCGGGTGAT
DSP_c.C4372G	CCCGAGAAAACAGGAGCTTATC	TTCCGGTCATTTGTTTCTTTG
DSP_c.G5167C	GCGATAGAAGATAAAAGCAGAAGC	CTGCAGCTGGCTCCTTAGTT
PPL_c.C322T	AAAACCATCAGGACCACGAC	GGATGCTGTGATTGTCTGGA
PPL_c.G2744T	CTCCTTGACCACGTACTCCTG	AGCCGGAAGTAGAAGTGACC
PPL_c.G1894A	CAGAGAAGGACTCCCAGGAC	CTGCAGCTGCTGGACTTG
PPL_c.A3152T	CTGCTTCTCCCTGAGCTG	GCGGATGAGGTCTTGCAG
PPL_c.C3460G	TTCTCCATCTCAGGGTCAGTC	TGCTCAAGGTGGAGAAGGAC
PPL_c.G3487A	TCCAGCTCACTCTGGTAGCTC	CTTCCTCCAGGACAAGCTCA
PPL_c.T4130A	GCGTATGGGTTACCTTCTCA	GAGCTTCCCAGGAAGAGCA

Supplementary Table 11. Taqman primers used for qPCR and SNP genotyping

qPCR			
**Gene symbol**	**Assay ID**	**Gene symbol**	**Assay ID**
DSP	Hs00189422_m1	*GCNT3*	Hs00191070_m1
PPL	Hs00160312_m1	*GLDC*	Hs01580586_g1
CAPN14	Hs00871882_m1	*GPR160*	Hs01878570_s1
TSLP	Hs01572934_g1	*GRK5*	Hs00992173_m1
GAPDH	Hs03929097_g1	*GRPEL2*	Hs00537120_s1
ACPP	Hs00173475_m1	*GYS2*	Hs00608677_m1
ACTG2	Hs01123712_m1	*H19*	Hs00262142_g1
ALOX12	Hs00167524_m1	*HILPDA*	Hs00203383_m1
ALOX15	Hs00609608_m1	*HPGDS*	Hs00183950_m1
ANO1	Hs00216121_m1	*HRH1*	Hs00911670_s1
APOBEC3A	Hs00377444_m1	*IGFL1*	Hs01651089_g1
ARG1	Hs00968979_m1	*IL13*	Hs01124272_g1
CA2	Hs00163869_m1	*IL4*	Hs00174122_m1
CCL26	Hs00171146_m1	*IL5*	Hs00174200_m1
CCR3	Hs99999027_s1	*IL5RA*	Hs00236871_m1
CD200R1	Hs00708558_s1	*JCHAIN*	Hs00950678_g1
CDA	Hs00156401_m1	*KCNJ2*	Hs01876357_s1
CDH26	Hs00375371_m1	*KRT23*	Hs00210096_m1
CFB	Hs00156060_m1	*LRRC31*	Hs00226845_m1
CFI	Hs00989715_m1	*MMP12*	Hs00899668_m1
CHL1	Hs00544069_m1	*MT1M*	Hs00828387_g1
CITED2	Hs01897804_s1	*MUC4*	Hs00366414_m1
CLC	Hs00171342_m1	*NEFL*	Hs00196245_m1
CLDN10	Hs01075312_m1	*NEFM*	Hs00193572_m1
CMA1	Hs00156558_m1	*PHLDB2*	Hs00377503_m1
COL8A2	Hs00697025_m1	*PMCH*	Hs00173595_m1
CPA3	Hs00157019_m1	*PNLIPRP3*	Hs00406604_m1
CRISP2	Hs00162960_m1	*POSTN*	Hs00170815_m1
CRISP3	Hs00195988_m1	*PTGFRN*	Hs01385989_m1
CRYM	Hs00157121_m1	*RTP4*	Hs00223342_m1
CTNNAL1	Hs00972098_m1	*RUNX2*	Hs00298328_s1
CTSC	Hs00175188_m1	*SAMSN1*	Hs00223275_m1
CXCL1	Hs00236937_m1	*SLC16A6*	Hs00190779_m1

CXCL6	Hs00237017_m1	*SLC26A4*	Hs01070620_m1
CXCL8	Hs01553824_g1	*SPINK7*	Hs00261445_m1
DSG1	Hs00355084_m1	*SUSD2*	Hs00219684_m1
EML1	Hs00270014_m1	*TNFAIP6*	Hs01113602_m1
ENDOU	Hs00195731_m1	*TPSB2*	Hs02576518_gH
EPB41L3	Hs00202360_m1	*TSPAN12*	Hs01113125_m1
EPPK1	Hs02379935_s1	*UBD*	Hs00197374_m1
F3	Hs01076032_m1	*UPK1A*	Hs01086736_m1
FCGR3A	Hs00275547_m1	*UPK1B*	Hs00199583_m1
FKBP5	Hs00296750_s1		Hs00209000_m1
FLG	Hs00863478_g1		
**SNP genotyping**			
CAPN14 (rs10192210)		C___8374570_20	

qPCR, quantitative polymerase chain reaction; SNP, single-nucleotide polymorphism.
