# Supplementary material for: Desmoplakin and periplakin genetically and functionally contribute to eosinophilic esophagitis
Source: Nat Commun. 2021 Nov 23;12:6795. doi: 10.1038/s41467-021-26939-9 (PMC8611043; doi:10.1038/s41467-021-26939-9)
Supplement: Supplementary file 2 — Reporting Summary [file 41467_2021_26939_MOESM2_ESM.pdf]

Corresponding author(s): Marc E. Rothenberg
Last updated by author(s): Sep 23, 2021

Reporting Summary

Nature Research wishes to improve the reproducibility of the work that we publish. This form provides structure for consistency and transparency in reporting. For further information on Nature Research policies, see our Editorial Policies and the Editorial Policy Checklist.

Statistics

For all statistical analyses, confirm that the following items are present in the figure legend, table legend, main text, or Methods section.

n/a	Confirmed	
☐	☒	The exact sample size (n) for each experimental group/condition, given as a discrete number and unit of measurement
☐	☒	A statement on whether measurements were taken from distinct samples or whether the same sample was measured repeatedly
☐	☒	The statistical test(s) used AND whether they are one- or two-sided<br>*Only common tests should be described solely by name; describe more complex techniques in the Methods section.*
☐	☒	A description of all covariates tested
☐	☒	A description of any assumptions or corrections, such as tests of normality and adjustment for multiple comparisons
☐	☒	A full description of the statistical parameters including central tendency (e.g. means) or other basic estimates (e.g. regression coefficient) AND variation (e.g. standard deviation) or associated estimates of uncertainty (e.g. confidence intervals)
☐	☒	For null hypothesis testing, the test statistic (e.g. F, t, r) with confidence intervals, effect sizes, degrees of freedom and P value noted<br>*Give P values as exact values whenever suitable.*
☒	☐	For Bayesian analysis, information on the choice of priors and Markov chain Monte Carlo settings
☒	☐	For hierarchical and complex designs, identification of the appropriate level for tests and full reporting of outcomes
☐	☒	Estimates of effect sizes (e.g. Cohen's d, Pearson's r), indicating how they were calculated

Our web collection on statistics for biologists contains articles on many of the points above.

Software and code

Policy information about availability of computer code

Data collection	Sequencing reads were aligned using Burrows-Wheeler Aligner (BWA) and GRCh37 human reference genome, and variant calls were made simultaneously following the Genome Analysis Toolkit (GATK) Best Practices by using the GATK Unified Genotyper.
Data analysis	JMP v13.1 (SAS Institute, Cary, NC), R statistical computing environment (version 3.1.2), GeneSpring GX 12.6 (Agilent Technologies, Santa Clara, CA), and GraphPad Prism 8 (GraphPad Software, Inc., San Diego, CA), Image Studio software 5.2(LI-COR Biosciences), MRI Wound Healing Tool (http://dev.mri.cnrs.fr/projects/imagej-macros/wiki/Wound_Healing_Tool), SnapGene 5.1(GSL Biotech), and Image J (NIH, Java 1.8.0_112 [64-bit]).

For manuscripts utilizing custom algorithms or software that are central to the research but not yet described in published literature, software must be made available to editors and reviewers. We strongly encourage code deposition in a community repository (e.g. GitHub). See the Nature Research guidelines for submitting code & software for further information.

Data

Policy information about availability of data

All manuscripts must include a data availability statement. This statement should provide the following information, where applicable:
- Accession codes, unique identifiers, or web links for publicly available datasets
- A list of figures that have associated raw data
- A description of any restrictions on data availability

The patients' genome data obtained by whole-exome sequencing are not publicly available owing to consent restrictions. The esophageal molecular expression data by EDP and single-cell RNA sequencing have been deposited in EGIDExpress (https://egidexpress.research.cchmc.org/data/). All other data supporting the findings of this study are included within the article or the Supplementary Information and are available from the corresponding author upon request.

Field-specific reporting

Please select the one below that is the best fit for your research. If you are not sure, read the appropriate sections before making your selection.

☒ Life sciences ☐ Behavioural & social sciences ☐ Ecological, evolutionary & environmental sciences

For a reference copy of the document with all sections, see nature.com/documents/nr-reporting-summary-flat.pdf

Life sciences study design

All studies must disclose on these points even when the disclosure is negative.

Sample size	All available human samples from patient families were used, and the sample size from families was not pre-determined. For in vitro studies, sample sizes were chosen to have minimum of a technical duplicate for an experiment. Individual experiments were repeated multiple times as indicated to ensure robustness and reproducibility.
Data exclusions	No data were excluded or representative data were shown where indicated.
Replication	All experiments were repeated multiple times as indicated. The number of successful replicas is indicated in figures legends, as a number experiments were repeated.
Randomization	No data for which randomization is required was used for this manuscript.
Blinding	mRNA expression and histologic assessments were all performed by observers blinded to genotype. Genotype was not revealed until all analyses were complete. The investigators were blinded to group allocation during data collection. For experiments other than mRNA and histologic experiments, no data for which blinding is required was used for this manuscript.

Reporting for specific materials, systems and methods

We require information from authors about some types of materials, experimental systems and methods used in many studies. Here, indicate whether each material, system or method listed is relevant to your study. If you are not sure if a list item applies to your research, read the appropriate section before selecting a response.

Materials & experimental systems

n/a	Involved in the study	
☐	☒	Antibodies
☐	☒	Eukaryotic cell lines
☒	☐	Palaeontology and archaeology
☒	☐	Animals and other organisms
☐	☒	Human research participants
☒	☐	Clinical data
☒	☐	Dual use research of concern

Methods

n/a	Involved in the study	
☒	☐	ChIP-seq
☒	☐	Flow cytometry
☒	☐	MRI-based neuroimaging

Antibodies

Antibodies used	Immunofluorescence: Anti-DSP (sc-390975, mouse monoclonal IgG1 A-1, Santa Cruz), Anti-PPL (ab131269, Rabbit monoclonal EPR8296, Abcam), Donkey anti-Mouse IgG Secondary Antibody, Alexa Fluor 488 (A32766, Invitrogen), Donkey anti-Rabbit IgG Secondary Antibody, Alexa Fluor Plus 647 (A32795, Invitrogen)<br>Western blotting: Anti-DSP (MAB9080, Monoclonal Mouse IgG1 Clone # 824038, R&D Systems), Anti–PPL (ab131269, Rabbit monoclonal EPR8296, Abcam), Anti-GAPDH antibodies (ab181602, Abcam; TA802519, Origene), Anti-FLAG (F3165, Sigma-Aldrich), Anti-HSP90 antibodies (ab13495, Abcam; TA500494, Origene), IRDye 680RD Goat anti-Mouse IgG Secondary Antibody (926-68070, LI-COR Biosciences), IRDye 800CW Goat anti-Rabbit IgG Secondary Antibody (926-32211, LI-COR Biosciences)
Validation	All antibodies were commercially available and used for their intended application (assay and species). All antibodies were used according to the dilution recommendations and application validated by manufacturer.

Eukaryotic cell lines

Policy information about cell lines

Cell line source(s)	EPC2 (hTERT-immortalized human esophageal epithelial cell line); gift from Dr. Anil Rustgi (University of Pennsylvania; currerntly AR is affiliated with Columbia University)<br>HaCaT (immortalized human skin keratinocyte line); gift from Dr. T. Bowden, University of Arizona, Tucson, AZ<br>HEK293T (human embryonic kidney cell-line 293T); ATCC

Authentication	Cell lines were authenticated by fingerprinting and visual inspection and carefully maintained in a centralized cell bank.
Mycoplasma contamination	All cell lines were tested routinely for mycoplasma contamination.
Commonly misidentified lines (See ICLAC register)	No commonly misidentified cell lines were used.

Human research participants

Policy information about studies involving human research participants

Population characteristics	Affected or unaffected individuals were recruited from Cincinnati Children's Hospital Medical Center (CCHMC), Cincinnati, USA: Familial EoE (62 multiplex families), non-familial EoE (511 non-multiplex families), and controls (93 individuals). The covariate-relevant population characteristics of the human research participants were detailed in supplementary information (Supplementary Table 8).
Recruitment	All patients were being seen for the clinical indication of eosinophilic esophagitis (EoE). All families were ascertained through a proband with EoE. No ascertainment nor recruitment bias was imposed on the basis of gender, ethnic origin, or any other demographic parameter. All samples and research protocols were collected in compliance with the Institutional Review Board at Cincinnati Children's Hospital Medical Center (CCHMC) after informed consent was obtained.
Ethics oversight	This study involving human subjects was conducted under an approved Institutional Review Board (Cincinnati Children's Hospital Medical Center) protocol number 2008-0090.

Note that full information on the approval of the study protocol must also be provided in the manuscript.
